# Type 2 Diabetes Is Associated with Altered NF-κB DNA Binding Activity, JNK Phosphorylation, and AMPK Phosphorylation in Skeletal Muscle after LPS

**DOI:** 10.1371/journal.pone.0023999

**Published:** 2011-09-13

**Authors:** Anne Sofie Andreasen, Meghan Kelly, Ronan Martin Griffin Berg, Kirsten Møller, Bente Klarlund Pedersen

**Affiliations:** 1 Centre of Inflammation and Metabolism, Department of Infectious Diseases and CMRC, University Hospital Rigshospitalet, Copenhagen, Denmark; 2 Intensive Care Unit 4131, University Hospital Rigshospitalet, Copenhagen, Denmark; 3 Intensive Care Unit, Department of Anaesthesiology, University Hospital Bispebjerg, Copenhagen, Denmark; University of Las Palmas de Gran Canaria, Spain

## Abstract

Systemic inflammation is often associated with impaired glucose metabolism. We therefore studied the activation of inflammatory pathway intermediates that interfere with glucose uptake during systemic inflammation by applying a standardised inflammatory stimulus *in vivo*. After ethical approval, informed consent and a thorough physical examination, 10 patients with type 2 diabetes and 10 participants with normal glucose tolerance (NGT) were given an intravenous bolus of *E. coli* lipopolysaccharide (LPS) of 0.3 ng/kg. Skeletal muscle biopsies and plasma were obtained at baseline and two, four and six hours after LPS. Nuclear factor (NF)-κB p65 DNA binding activity measured by ELISA, tumor necrosis factor-α and interleukin-6 mRNA expression analysed by real time reverse transcription polymerase chain reaction, and abundance of inhibitor of NF-κB (IκB)α, phosphorylated c-Jun-N-terminal kinase (JNK), AMP-activated protein kinase (AMPK), and acetyl-CoA carboxylase measured by Western blotting were detected in muscle biopsy samples. Relative to subjects with NGT, patients with type 2 diabetes exhibited a more pronounced increase in NF-κB binding activity and JNK phosphorylation after LPS, whereas skeletal muscle cytokine mRNA expression did not differ significantly between groups. AMPK phosphorylation increased in volunteers with NGT, but not in those with diabetes. The present findings indicate that pathways regulating glucose uptake in skeletal muscle may be involved in the development of inflammation-associated hyperglycemia. Patients with type 2 diabetes exhibit changes in these pathways, which may ultimately render such patients more prone to develop dysregulated glucose disposal in the context of systemic inflammation.

**Trial Registration:**

ClinicalTrials.gov NCT00412906

## Introduction

Type 2 diabetes is associated with low-grade systemic inflammation [1]–[4], and increased levels of circulating proinflammatory mediators, e.g. tumour necrosis factor-alpha (TNFα) and gut-derived lipopolysaccharide (LPS), may play a role in the pathogenesis of insulin resistance, the hallmark of type 2 diabetes [5]–[8]. In diabetic patients as well as in non-diabetic persons, the binding of TNFα, LPS or other pro-inflammatory ligands to their membrane-bound receptors, activates intracellular signaling pathways that facilitate the dissociation of nuclear factor (NF)-κB from inhibitor of NF-κB (IκB) proteins [9], [10]. NF-κB subsequently enhances the transcription of a vast array of genes encoding inflammatory mediators, e.g. TNFα and interleukin (IL)-6 [11]–[13]. Apart from inducing further NF-κB activity, the concurrent increase in circulating TNFα may mediate the phosphorylation and activation of the intracellular signaling molecule c-Jun-N-terminal kinase (JNK) in skeletal muscle [5]. Phosphorylated JNK (p-JNK) induces serine phosphorylation of insulin receptor substrate (IRS)-1, which inhibits insulin signal transduction [14]–[17]. Thus, excessive and inappropriate activation of NF-κB and JNK in skeletal muscle may lead to insulin resistance. Accordingly, the NF-κB pathway and JNK activities have been found to be increased in skeletal muscle of patients with type 2 diabetes [18]–[20].

Systemic inflammation may also affect non-insulin dependent pathways regulating glucose disposal. AMP-activated protein kinase (AMPK) is a fuel-sensing enzyme present in all mammalian cells, which upon phosphorylation and subsequent activation regulates glucose uptake in skeletal muscle by increasing GLUT4 translocation via signaling pathways that are distinct from those of insulin [21], [22]. AMPK may link non-insulin dependently regulated glucose disposal in skeletal muscle to inflammatory signaling, since TNFα seems to inhibit the activation of AMPK [23]. However, the reports on AMPK activity in the skeletal muscle in type 2 diabetes have so far been ambiguous [24]–[26]. Although it is well known that circulating proinflammatory mediators induce insulin resistance in skeletal muscle [5], it is presently unknown how a standardized inflammatory stimulus affects the activity of NF-κB, JNK and AMPK in skeletal muscle of diabetic patients. Aberrations in the inflammation-induced responses of these central intermediates may potentially contribute to the disturbances in glucose disposal observed in diabetic patients.

In order to perform a comparative investigation of intra-muscular changes in inflammatory intermediates during standardised conditions, we subjected patients with type 2 diabetes and subjects with normal glucose tolerance to the human endotoxin model [27]. We hypothesised that the inflammatory stimulus, an intravenous bolus injection of *Escherichia coli* LPS, would increase the activity of intermediates associated with insulin resistance, i.e. NF-κB and p-JNK, more profoundly in patients with type 2 diabetes, while the phosphorylation of AMPK was expected to be diminished.

## Materials and Methods

### Ethics statement

The Ethical Committee of Copenhagen and Frederiksberg Municipalities approved the study protocol (KF 01-320695). This study was conducted according to the guidelines laid down in the Declaration of Helsinki, written informed consent was obtained from all subjects, and the trial was registered at www.clinicaltrials.gov (NCT 00412906). The protocol for this trial and supporting CONSORT checklist are available as supporting information; see Checklist S1, Flowchart S1 and Protocol S1.

### Subjects

The study was carried out between November 2006 and July 2009. Ten subjects with normal glucose tolerance (NGT) and 10 age-matched patients with type 2 diabetes were selected from a larger cohort of diabetic or non-diabetic males, who had all been subjected to LPS injection [28]. The inclusion age was from 18 to 80 years. Type 2 diabetes was defined by the WHO classification criteria [29], in which glucose tolerance was determined by an oral glucose tolerance test (OGTT). Exclusion criteria were symptoms or a medical history of cardiovascular, pulmonary, renal or autoimmune diseases, as well as treatment with insulin, systemic anti-inflammatory drugs, anti-coagulants, ACE-inhibitors or angiotensin II-antagonists. Of the 10 patients with diabetes, six were medically treated for their diabetes. Three received treatment with metformin, two with sulfonylurea, one with thiazoledinediones, and three with statins, either as single-drug or combination therapy. All-diabetes-related medications, including statins, were withheld for seven days prior to the study. No volunteer experienced fever or any symptoms of infection during the fortnight preceding the study.

#### Endotoxin injection

**Figure 1:**
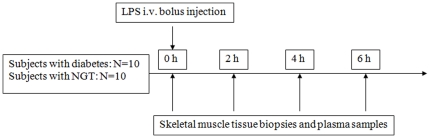
Schematic overview of the study design. Fig. 1 10 patients with diabetes and 10 volunteers with NGT were included in the study. After obtaining skeletal muscle biopsies and plasma samples at baseline (0 h), subjects received an intravenous bolus injection of LPS, 0.3 ng/kg. Muscle tissues biopsies and plasma samples were also obtained at 2, 4 and 6 hours after LPS. NGT: Normal glucose tolerance, LPS: lipopolysaccharide.

The endotoxin model has been described previously [30]. After an overnight fast, volunteers reported to the Intensive Care Unit, where they were bed-rested and remained fasting for the entire study day. Intravenous catheters were inserted into cubital veins bilaterally and, after obtaining baseline skeletal muscle biopsies and blood samples, an intravenous bolus injection of *E. coli* LPS of 0.3 ng/kg was administered. Additional blood samples and biopsies were obtained two, four and six hours after the LPS injection. Blood samples were spun at 3,500*g for plasma isolation. Plasma was stored at −80°C until the later measurement of TNFα and IL-6. The study design is schematically presented in **Figure 1** ****.

#### Biopsies

After anesthetising the skin with 2 ml Lidocain 2%, ∼100 mg of skeletal muscle tissue was extracted with a Bergström biopsy needle under sterile conditions. The tissue samples were rinsed with sterile saline, and any visible vasculature was removed. The samples were then immediately frozen in liquid nitrogen and stored at −80°C until analysis. Skeletal muscle tissue from the 10 + 10 subjects was analysed en bloc for NF-κB p65 DNA binding activity (ELISA), phosphorylated and total JNK, phosphorylated AMPK (p-AMPK) and total AMPK (Western blot), as well as TNFα and IL-6 mRNA expression (PCR). In order to confirm our findings on NF-κB binding activity, biopsies were analysed for the abundance of the primary inhibitor of NF-κB (IκB)α (Western blot), while the phosphorylated and the total amount of acetyl-CoA carboxylase (ACC), a downstream target molecule of AMPK was measured as a control of our AMPK analyses (Western blot).

#### Plasma analyses

Plasma levels of TNFα and IL-6 were determined in duplicate, using ELISA (R&D Systems, Minneapolis, MN, USA). The effect of inter-assay variation was minimised by performing the time-consecutive measurements of each subject as well as analysing matched pairs of patients with type 2 diabetes and subjects with NGT on the same ELISA plates. Intra-assay variation was evaluated by the coefficient of variation (CV) of the duplicate measurements. The duplicate TNFα and IL-6 measurements had a CV above 20% in 1% and 2.3% of the measurements, respectively, and were therefore excluded from the subsequent analyses. Of the remaining measurements, the mean intra-assay variation was 4.5% in the TNFα measurements and 5.4% in the IL-6 measurements.

#### RNA isolation, reverse transcription and real-time polymerase chain reaction (PCR)

Total RNA was extracted from the muscle biopsy by means of the following procedure: Skeletal muscle tissue was homogenised in 1 ml Trizol Reagent (Invitrogen, Carlsbad, CA, USA) for 15 seconds using a Qiagen Tissuelyser (Qiagen Nordic, Copenhagen, Denmark). Chloroform was then added and the phases separated by centrifugation. The RNA-containing aqueous phase was transferred to a fresh tube and incubated with isopropanol at room temperature for 10 minutes for the precipitation of RNA. After another centrifugation, the RNA pellet was washed in 75% ethanol and finally dissolved in 15 µl Ultrapure DNAse/RNAse-Free Distilled Water (Invitrogen, Carlsbad, CA, USA).

The RNA concentration was determined spectrophotometrically on a NanoDrop 1000 spectrophotometer (Thermo Fisher Scientific, Waltham, MA, US) and 5 µg total RNA was reversed-transcribed in a total volume of 200 µl, using the High Capacity cDNA Reverse Transcription kit (Applied Biosystems, Branchburg, NJ, US) and run on a GeneAmp^®^ PCR System 9700 (Applied Biosystems, Branchburg, NJ, US). Real-time PCR was performed using an ABI 7900 Sequence Detection System (Applied Biosystems, Branchburg, NJ, US). TNFα and IL-6 cDNA were amplified using molecular primer/probe assays with the TaqMan® Universal PCR Master Mix (Applied Biosystems, Branchburg, NJ, US). Primer and probe sequences were as follows: TNFα: forward 5′- GGAGAAGGGTGACCGACTCA-3′; reverse: 5′- TGCCCAGACTCGGCAAAG-3′; Probe: FAM-5′- CGCTGAGATCAATCGGCCCGACTA-3′-TAMRA; IL-6: forward 5′- CCAGGAGCCCAGCTATGAAC-3′; reverse: 5′- CCCAGGGAGAAGGCAACTG-3′; Probe: FAM-5′-CCTTCTCCACAAGCGCCTTCGGT-3′-TAMRA. Primers and probe for 18S ribosomal RNA were pre-developed Taqman assay from Applied Biosystems, Branchburg, NJ, US (catalog # 4310893E). Primer pair efficiencies were determined and primer/probe concentrations were optimised in the validation of each primer set. The threshold cycle (Ct) value for 18S was subtracted from the Ct value for the target gene (obtaining ΔCt) to adjust for any variations in the cDNA synthesis, and the 2^−ΔΔCT^ method [31] was used to calculate the relative changes in mRNA abundance.

#### Skeletal muscle lysate

Skeletal muscle lysate was prepared by mixing the muscle tissue with cell lysis buffer A (containing 30 mM Hepes pH 7.4, 2.5 mM EGTA, 3 mM EDTA, 20 mM KCl, 40 mM β-glycerol phosphate, 40 mM NaF, 4 mM NaPPi, 1 mM Na_3_VO_4_, 32% Glycerol, 0.1% Ipegal CA-630, containing Phosphatase Inhibitor Cocktail 1 and 2 (Sigma-Aldrich, St. Louis MO, USA) and a complete protease inhibitor cocktail (Roche, Basel, Switzerland)), followed by homogenisation in precooled racks using a tissue lyser (Qiagen, Valencia, CA, USA) for 1 min at 30 Hz and 5 min incubation on ice. Homogenisation and incubation were repeated two or three times to obtain the required degree of homogenisation. Homogenates were then centrifuged at 16,000*g at 4°C for 10 min. The supernatant was transferred to a new tube and an equal amount of cell lysis buffer B (similar to buffer A with the following exceptions: 70 mM KCl, 20 mM β-glycerol phosphate, 20 mM NaF, 2 mM NaPPi, 0% glycerol) was added. The resulting protein lysates were centrifuged at 16,000*g and 4°C for 10 min and the supernatant transferred to a new tube. Protein concentrations were measured using the Bio-Rad DC kit (Bio-Rad, Hercules, CA, USA) using BSA as standard. All measurements were done in triplicate.

#### Western blot

Aliquots of lysate from skeletal muscle corresponding to 25 µg of total protein were electrophoresed in 4–12% polyacrylamide gradient gels and then transferred onto polyvinylidene fluoride (PVDF) membranes. The membranes were blocked with 5% milk in Tris-buffered saline (pH 7.5) containing 0.05% Tween 20 (TBST) for 1 h at room temperature. The blots were first incubated overnight with primary antibody, including anti-p-AMPKα-Thr^172^, anti-AMPKα, anti-p-ACC-ser^79^, anti-p-JNK (recognizing phosphorylation of the 46- and 54-kDa isoforms of JNK at Thr^183^ and Tyr^185^), anti-JNK, anti-IκBα (Cell Signaling, Danvers, MA, USA) or anti-ACCβ (Santa Cruz Biotechnology, Santa Cruz, CA, USA). Primary antibodies were diluted 1∶1,000 in TBST containing 5% BSA. The blots were then incubated in TBST containing 5% nonfat dry milk and the appropriate secondary antibody conjugated to horseradish peroxidase at a 1∶5,000 dilution. Each membrane was afterwards stained with Reactive brown in order to ensure sufficient blotting, even protein loading and to achieve a measure of total protein content in each lane.

In order to minimize bias from inter-assay variation, gels including only baseline samples were run for the comparison of baseline values of diabetic patients and healthy controls. Furthermore, time consecutive samples (0, 2, 4, 6 h post LPS) of each subject were run on the same gel, and each gel included samples from participants with NGT as well as from patients with diabetes. Bands were detected using Supersignal West Femto (Pierce, Rockford, IL, USA) and quantified using a CCD image sensor (ChemiDocXRS, Bio-Rad) and software (Quantity One, Bio-Rad). The two bands of p-JNK / JNK (46 and 54 kDA) were quantified together.

All intensities of IκBα, p-JNK, total JNK, p-AMPKα, total AMPKα, p-ACC and total ACCβ were divided by those from the Reactive brown staining of the corresponding membranes. Abundance of IκBα and p-JNK in the time-consecutive measurements are expressed as arbitrary units to Reactive brown. Baseline p-JNK and all measurements of p-AMPK and p-ACC are expressed as arbitrary units to the total JNK, AMPK and ACC, respectively. Fold increases after LPS was calculated with each subject serving as his own control.

#### NF-κB p65 DNA-binding activity

DNA-binding activity of the p65 NF-κB subunit was measured in protein lysates using an enzyme-linked immunosorbent assay (ELISA) kit (Active Motif, Rixensart, Belgium) according to the manufacturer's instructions. Activity at baseline is expressed as absorbance at 490 nm; fold increases after LPS was calculated with every subject serving as his own control.

#### Statistical analysis

Subject characteristics (age, body mass index (BMI), fasting glucose, fasting insulin, HbA1c, NF-κB DNA binding activity, and IκBα abundance, p-JNK, p-AMPK and p-ACC) are presented as mean and standard deviation (SD). Plasma cytokine concentrations measurements were logarithmically transformed prior to statistical analysis and the estimated mean and 95% confidence intervals (95% CI) transformed back to the original scale, providing geometric means for the presentation of results. TNFα and IL-6 mRNA expression at baseline is given as the ratio between ΔCt of patients with diabetes to that of volunteers with NGT.

Between-group comparisons (patients with diabetes vs. participants with NGT) at baseline were performed with a one-way ANOVA. Within-subject and between-group variations over time were analysed with two-way ANOVAs for longitudinal measurements (SAS 9.1 proc mixed), investigating the effect of time (hours post LPS), the effect of diabetes, and the effect of time in interaction with diabetes on the outcome variable. Models were fitted by backward regression, and goodness-of-fit was assessed by evaluating the distribution of the residuals. If the ANOVA indicated a significant effect of time, Dunnett-adjusted post-hoc t-tests were subsequently performed to identify significant differences from baseline. In case of missing data, we performed the statistical analyses without the measurements from the specific time points. The number of missing data points were minimal and restricted to a few sporadic Western blot analyses and ELISA analyses.

P<0.05 was considered to indicate statistical significance. SAS statistical software 9.1 was used for all statistical analyses.

## Results

The baseline characteristics and plasma cytokine concentrations of a larger cohort have been reported as part of a separate manuscript [28].

At baseline, patients with type 2 diabetes demonstrated increased plasma glucose and HbA1c when compared to subjects with NGT, while groups were similar with regard to age and BMI, fasting plasma insulin, and NF-κB p65 DNA binding activity, IκBα abundance, p-AMPK, p-ACC, and TNFα and IL-6 mRNA expression in skeletal muscle (**Table 1**). Patients with type 2 diabetes exhibited a trend towards higher p-JNK at baseline (P = 0.09).

**Table 1:** Baseline characteristics.

	*NGT (n = 10)*		*Type 2 diabetes (n = 10)*		*P-value*
	*Mean (SD)*	*Range*	*Mean (SD)*	*Range*	
**Age (years)**	64 (11)	(39–78)	54 (15)	(32–73)	NS
**BMI (kg/m^2^)**	27.6 (5.2)	(22.9–38.8)	28.5 (8.3)	(22.3–48.3)	NS
**Fasting glucose (mmol/l)**	5.5 (0.8)	(4.7–6.3)	12.1 (4.1)	(7.1–23.1)	0.002
**HbA1c (mmol/l)**	5.4 (0.4)	(4.6–5.8)	8.6 (3.0)	(6.0–13.4)	0.01
**Fasting insulin (pmol/l)**	56 (23)	(29–105)	70 (26)	(33–125)	NS
**NF-κB p65 DNA-binding activity (absorbance)**	0.23 (0.12)		0.17 (0.07)		NS
**IκBα (arbitrary units)**	1.5 (1.2)		2.0 (1.3)		NS
**p-JNK (arbitrary units)**	0.3 (0.1)		0.4 (0.2)		NS *
**p-AMPK (arbitrary units)**	1.1 (0.4)		0.9 (0.2)		NS
**p-ACC (arbitrary units)**	0.3 (0.14)		0.2 (0.1)		NS
	***Mean (SD)***				***P-value***
**TNFα mRNA content ** ***(Ratio, Diabetes vs. NGT)***	1.0 (0.5)				NS
**IL-6 mRNA content ** ***(Ratio, Diabetes vs. NGT)***	1.2 (0.7)				NS

Baseline characteristics of 10 patients with type 2 diabetes and 10 volunteers with NGT are presented as mean (age, BMI, HbA1c, HOMA2-IR, NF-κB activity) and standard deviation. Age, BMI, fasting glucose, HbA1c and fasting insulin are also represented with ranges. IκBα, p-JNK, p-AMPK and p-ACC are expressed as mean arbitrary units relative to Reactive brown (IκBα), total JNK, total AMPK, or total ACC, respectively.

*P = 0.09. SD: standard deviation, NS: Non-significant, BMI: Body mass index, TNFα: tumour necrosis factor-alpha, IL-6: interleukin-6, NF-κB: nuclear factor κB, IκBα: inhibitor of NF-κB α, p-JNK: phosphorylated c-Jun-N-terminal kinase, p-AMPK: phosphorylated AMP-activated protein kinase, p-ACC: phosphorylated acetyl-CoA carboxylase, NGT: normal glucose tolerance.

**Figure 2:**
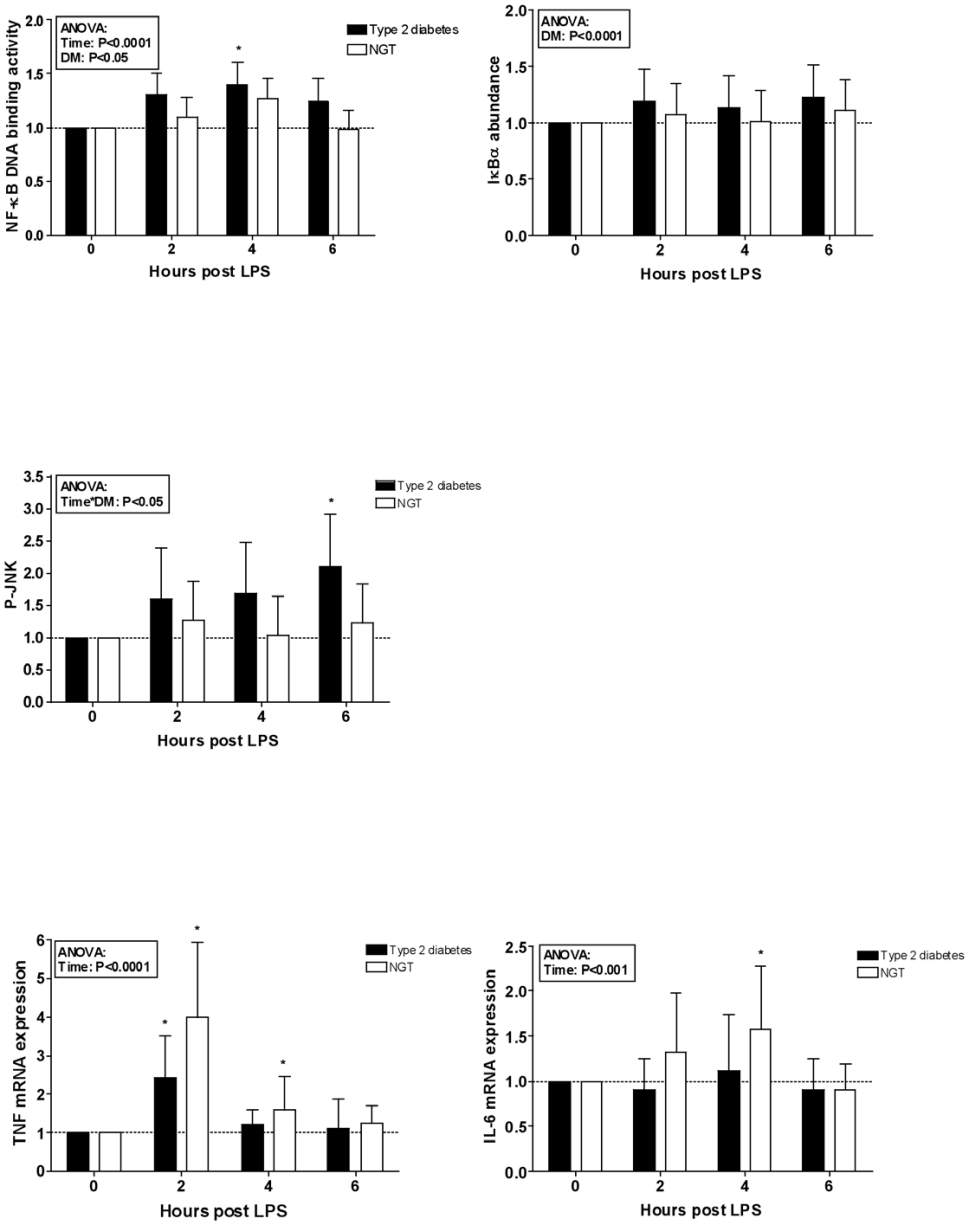
Fold increases in NF-κB, IκBα, p-JNK, TNFα mRNA and IL-6 mRNA after LPS. Fig. 2 Fold increases (mean and 95% confidence interval) from baseline following an intravenous injection of LPS in 10 patients with diabetes (▪) and 10 volunteers with normal glucose tolerance (□). * P<0.05 compared to baseline. LPS: lipopolysaccharide, NF-κB: nuclear factor κB, IκBα: inhibitor of NF-κB α, p-JNK: phosphorylated c-Jun-N-terminal kinase, TNFα: tumour necrosis factor-alpha, IL-6: interleukin-6.

**Figure 3:**
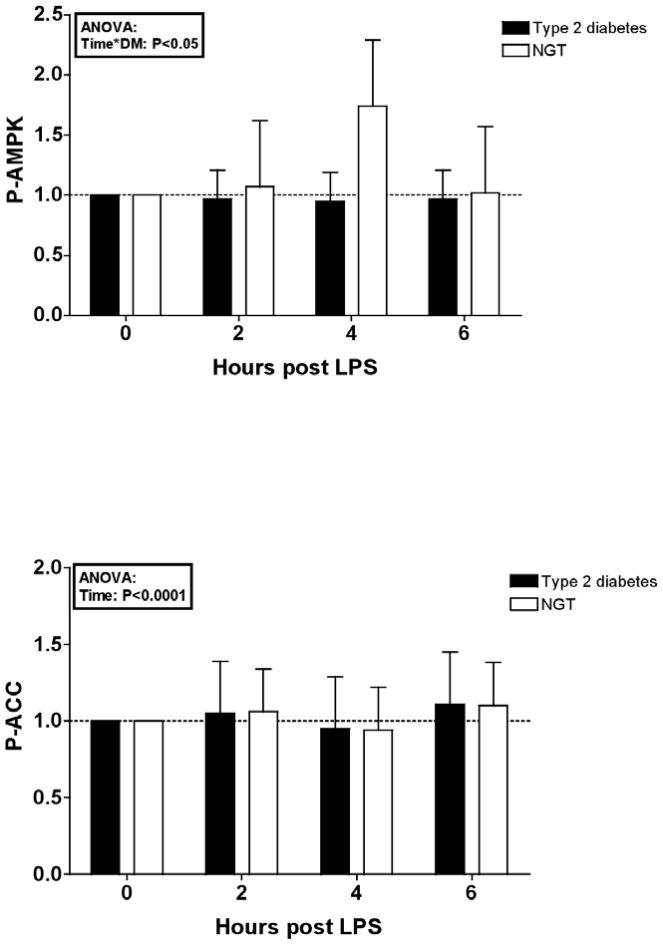
Fold increases in p-AMPK and p-ACC after LPS. Fig. 3 Fold increases (mean and 95% confidence interval) from baseline following an intravenous injection of LPS in 10 patients with diabetes (▪) and 10 volunteers with NGT (□). LPS: lipopolysaccharide, NGT: normal glucose tolerance, p-AMPK: phosphorylated AMP-activated protein kinase, p-ACC: phosphorylated acetyl CoA carboxylase.

Compared to baseline, LPS administration induced an increase in NF-κB p65 DNA binding activity, JNK phosphorylation and expression of TNFα and IL-6 mRNA in skeletal muscle (**Figure 2**). The NF-κB DNA binding activity was overall higher in type 2 diabetic patients when compared to NGT subjects (ANOVA, effect of diabetes: P<0.05). We expected a concurrent reduction in the abundance of the primary inhibitor of NF-κB, IκBα, as this would indicate a degradation of the IκB subunits subsequent to the separation from NF-κB. In contrast, levels of IκBα were slightly, but highly significantly increased in patients with diabetes compared to subjects with NGT (ANOVA, effect of diabetes: P<0.0001). Increases in p-JNK were more pronounced among patients with diabetes (ANOVA, effect of interaction between diabetes and time: P<0.05), while the expression of TNFα and IL-6 mRNA did not differ significantly between diabetic and non-diabetic volunteers. p-AMPK increased in subjects with NGT, while this response was blunted in diabetic patients (ANOVA, effect of interaction between diabetes and time: P<0.05) (**Figure 3**). However, this difference was not reflected in the phosphorylation of ACC, a downstream target molecule of AMPK.

LPS induced a systemic inflammatory response with increases in plasma TNFα and IL-6 in both diabetic and non-diabetic volunteers (**Table 2**). There was a trend towards lower plasma TNFα response during endotoxemia in patients with diabetes (ANOVA, effect of diabetes: P = 0.07), while the two groups did not differ with regard to the IL-6 response.

**Table 2:** Plasma cytokines, glucose and free fatty acids before and after LPS administration.

		Hours post LPS injection			
		*0-h*	*2-h*	*4-h*	*6-h*
**TNFα (pg/ml) †**	**Type 2 diabetes**	1.0 (0.8–1.3)	17.7 * (13.6–22.9)	7.0 * (5.4–9.0)	3.2 * (2.4–4.1)
	**NGT**	1.3 (1.0–1.8)	24.5 * (18.4–32.8)	8.9 * (6.7–11.9)	3.8 * (2.8–5.1)
**IL**–**6 (pg/ml)**	**Type 2 diabetes**	1.7 (1.2–2.5)	55.4 * (38.4–80.0)	23.5 * (16.5–33.6)	5.9 * (4.1–8.6)
	**NGT**	1.6 (1.1–2.2)	50.0 * (34.7–73.0)	20.0 * (13.8–28.8)	4.8 * (3.3–7.0)

Plasma cytokines (geometric mean and 95% confidence interval) following an intravenous injection of LPS in 10 patients with diabetes and 10 volunteers with normal glucose tolerance. Statistical analyses and comparison were performed on the log-transformed measured concentrations, and the predicted means and 95% CI derived from the ANOVAs were then transformed back to the original scale for presentation. † P = 0.07 (effect of diabetes, two-way ANOVA).

*P<0.05 (compared to baseline measurement, Dunnett-adjusted post-hoc t-tests). No differences were detected between groups at individual time-points, LPS: lipopolysaccharide, TNFα: tumor necrosis factor-alpha, NGT: Normal glucose tolerance, IL-6: Interleukin 6.

## Discussion

The present study demonstrates that skeletal muscle tissue of patients with type 2 diabetes exhibits a different response to LPS than subjects with normal glucose tolerance with: 1) increased NF-κB p65 DNA binding activity, 2) increased JNK phosphorylation, and 3) blunted AMPK phosphorylation.

The association between systemic inflammation and aberrations in glucose metabolism in type 2 diabetes, is well described in the literature [3], [32]–[34]. The increased NF-κB p65 DNA binding activity and increased phosphorylation of JNK demonstrated in diabetic skeletal muscle in the present study align with previous studies, which functionally linked such changes to skeletal muscle insulin resistance [16]–[19]. Although the underlying mechanisms that prompted the accentuated LPS-induced changes in NF-κB and p-JNK in the diabetic patients in this study remain obscure, putative stimuli for the NF-κB and JNK pathways, such as elevated free fatty acids (FFA), advanced glycation end products, reactive oxygen species and endoplasmatic reticulum stress [19], [35]–[37], are frequently present in diabetic patients and may act in synergy with LPS to compound the effects on NF-κB and JNK activity. Previous studies demonstrate that both mRNA expression and protein content of the membrane-bound receptor of LPS, Toll-like receptor 4 (TLR4) [19], as well as NF-κB activity are elevated in unstimulated conditions in skeletal muscle of patients with type 2 diabetes. Although we did not detect higher levels of NF-κB binding activity at baseline in our diabetic volunteers, we cannot exclude the possibility that increased TLR4 signaling could account for the fold increases in NF-κB activity and JNK after endotoxin administration as observed in our study. The modest but significant LPS-induced increases in NF-κB activity and p-JNK among the normal glucose tolerant volunteers suggest that these pathways may also be involved in inflammation-associated insulin resistance, even in the absence of diabetes, for example in critical illness and sepsis.

Surprisingly, we detected an apparent temporal inconsistency between the measured peak values of the NF-κB activity and TNFα expression. According to the ANOVA, LPS-exposure elicited an overall time-dependent change both in NF-κB binding activity and in cytokine expression. However, TNFα expression appears to peak prior to the NF-κB activity (**Figure 2**). This is somewhat surprising, since NF-κB is an upstream activator of TNFα upon LPS-exposure. Alternative pathways that are independent of NF-κB may induce the early upregulation of TNFα expression. Apart from JNK, these pathways may involve LPS-induced activation of skeletal muscle MAP-kinases, such as p38 and ERK [38]. It is also puzzling that NF-κB activity is increased in patients with type 2 diabetes, while no significant overall differences in cytokine mRNA expression were detected between groups. This discrepancy may be explained by tolerance phenomena [39] or epigenetic changes [40], [41]; however the present study design did not allow us to investigate these mechanisms further. Finally, we cannot exclude the possibility that the variability associated with human studies and the relatively small sample sizes have affected the results.

The findings of a LPS-induced increase in NF-κB p65 DNA binding activity could not be confirmed by an accompanying decrease in the abundance of its primary inhibitor, IκBα. *In vitro* stimulation of fibroblasts with TNFα induces complex oscillations in IκBα levels with decreases and increases that vary within intervals as short as 30 minutes [9]. It is therefore likely that the degradation of IκBα was not detected due to the longer time span in between our samples being obtained, and that the nadir of IκBα abundance occurred between two measurements. Considering the previously detected fluctuations of IκBα, skeletal muscle biopsies should ideally have been obtained at intervals as short as 15 minutes; however, this would neither have been ethically nor practically possible.

Although skeletal muscle p-AMPK in volunteers with NGT and diabetes was comparable at baseline, the LPS-induced p-AMPK response was blunted in diabetic skeletal muscle. Previous studies have provided no clear picture of how diabetes affects skeletal muscle AMPK activity. Investigations during resting conditions have revealed conflicting results [24], [42], [43]. During exercise, obesity rather than diabetes seems to impact exercise-induced AMPK activity [44]. Furthermore, age but not diabetes was found to influence AMPK activation upon administration of AICAR, a pharmacological activator of AMPK, to diabetic and non-diabetic volunteers [45]. The two groups in the present study were comparable with regard to age and BMI; hence, the blunted LPS-induced p-AMPK response in diabetic skeletal muscle cannot be attributed to weight or age differences, but is likely attributable to additional factors, such as chronically elevated plasma glucose in diabetic patients, which may inhibit AMPK activation in a similar manner to what is observed during exercise [46].

Our findings in normal skeletal muscle are somewhat in contrast to a previous study by Fredriksson et al [26], in which an LPS injection at a dose of 4 ng/kg neither affected total AMPK or p-AMPK. This discrepancy is likely explained by differences in study designs, since only young and lean males with NGT were included in the previous study. Furthermore, AMPK phosphorylation is inhibited by TNFα [23], at least in non-diabetic skeletal muscle. The absent changes in p-AMPK in the study by Frederiksson et al may therefore have been caused by the immense increase in plasma TNFα that occur when LPS is administered at 4 ng/kg [47]; this contrasts to the relatively moderate TNFα increases (at 20-25 fold) that were observed after a bolus injection of 0.3 ng/kg in the present study.

It is surprising that the increases in AMPK phosphorylation were not accompanied by corresponding changes in the downstream target of AMPK, ACC. The lack of ACC phosphorylation may be due to changes in phosphorylation that occur between sampling time points. Corresponding changes in p-ACC would have increased the validity of the detected changes in p-AMPK among the normal glucose tolerant volunteers, and consequently the AMPK findings must be interpreted with caution.

Having studied the specified intermediates NF-κB, IκBα, TNFα, IL-6, JNK, AMPK and ACC, it is noteworthy that the study does not provide a complete picture of the connection between signaling pathways promoting inflammation and those regulating insulin sensitivity in the skeletal muscle tissue of type 2 diabetic patients. Investigating other targets with central placements in the insulin signaling cascade, e.g. insulin receptor substrate (IRS)-1 and Akt, would possibly provide additional important information in clarifying these connections. Unfortunately, the present study did not provide biopsy material enough for such analyses and future studies will have to address these topics.

In conclusion, we have demonstrated that subjects with NGT and patients with type 2 diabetes responded differently to the administration of LPS with regard to NF-κB p65 DNA binding activity, JNK phosphorylation and AMPK phosphorylation in skeletal muscle. The present findings provide evidence for an enhanced response of the NF-κB and JNK pathways in diabetic skeletal muscle upon an inflammatory insult; both may contribute to inflammation-associated insulin resistance. Additionally, patients with type 2 diabetes appear not to mount the same increases in p-AMPK as persons with NGT after an inflammatory response. The changes in inflammation-induced signaling in diabetic compared to non-diabetic skeletal muscle tissue may ultimately render these patients more prone to develop aberrations in skeletal muscle glucose disposal during systemic inflammation.

## Supporting Information

Checklist S1 **Consort Checklist**
(DOC)

Flowchart S1 (PDF)

Protocol S1 Protocol to the Ethical Committee of Copenhagen and Frederiksberg Municipalities approved the study protocol (KF 01-320695).
(DOC)
