# Supplementary figures and images for: Type 2 Diabetes Is Associated with Altered NF-κB DNA Binding Activity, JNK Phosphorylation, and AMPK Phosphorylation in Skeletal Muscle after LPS
Source: PLoS One. 2011 Sep 13;6(9):e23999. doi: 10.1371/journal.pone.0023999 (PMC3172218; doi:10.1371/journal.pone.0023999)

## CONSORT 2010 Flow Diagram

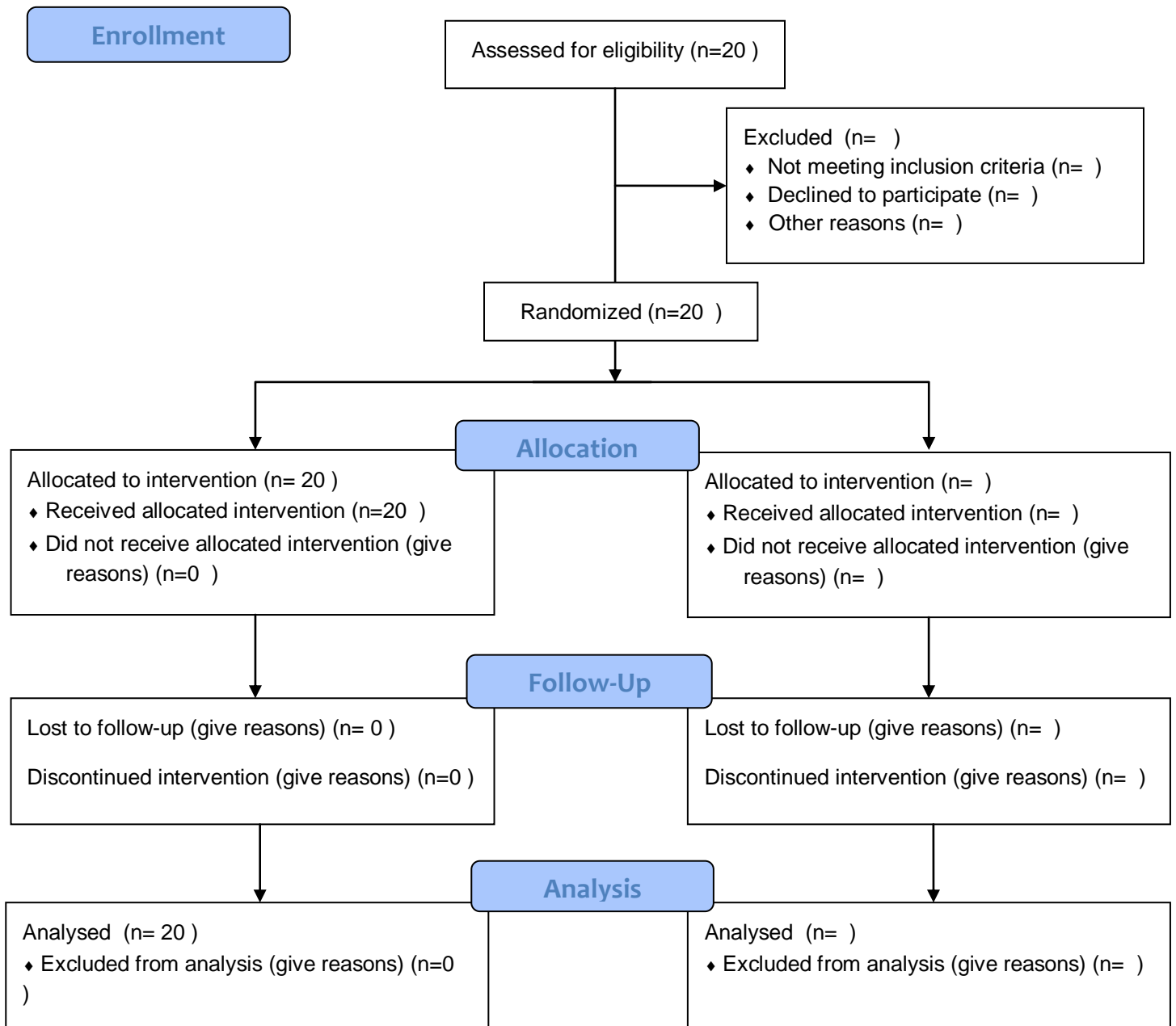

Supplement: Flowchart S1 — (PDF) [file pone.0023999.s002.pdf]
