# Supplementary material for: Type 2 Diabetes Is Associated with Altered NF-κB DNA Binding Activity, JNK Phosphorylation, and AMPK Phosphorylation in Skeletal Muscle after LPS
Source: PLoS One. 2011 Sep 13;6(9):e23999. doi: 10.1371/journal.pone.0023999 (PMC3172218; doi:10.1371/journal.pone.0023999)
Supplement: Protocol S1 — Protocol to the Ethical Committee of Copenhagen and Frederiksberg Municipalities approved the study protocol (KF 01-320695). (DOC) [file pone.0023999.s003.doc]

**Protokol:**

**Systemisk inflammation ved diabetes mellitus type II – betydning for renal hæmodynamik, insulinresistens og effekt af probiotiske bakterier.**

af

Læge Sofie Andreasen

Epidemiafdelingen, Center for Inflammation og Metabolisme

Rigshospitalet

*Øvrige deltagere*:

Jan Bonde, overlæge, dr. med., Intensiv afdeling 4131, Rigshospitalet (RH)

Bo Feldt-Rasmussen, overlæge, dr. med., Nefrologisk afdeling, RH

Henrik Gutte, læge, Klinisk Fysiologisk afdeling, RH

Mogens Jakobsen, professor, Kgl. Veterinær- og Landbohøjskole (KVL)

Andreas Kjær, professor, dr. med., Klinisk fysiologisk afdeling, RH

Nadja Larsen, levnedsmiddelkandidat, ph.d. stud., KVL

Kirsten Møller, 1. reservelæge, ph.d, Epidemiafdelingen, Center for Inflammation og Metabolisme, RH

Michael Lehd Møller, civ.ing., hospitalsfysiker, Klinisk Fyisologisk afd., Frederiksberg Hospital

Bente Klarlund Pedersen, professor, dr. med., Epidemiafdelingen, Center for Inflammation og Metabolisme, RH

Anders Perner, afdelingslæge, ph.d, Intensiv afdeling 4131, RH

**Formål**

Hos type 2 diabetikere og raske forsøgspersoner at måle

1. effekten af endotoxinæmi på genekspressionen i muskel- og fedtvæv af inflammatoriske og metabolismeregulerende signalstoffer;

2. effekten af endotoxinæmi på nyrernes blodtilførsel og den glomerulære filtrationsrate, bestemt ved diethylene triamine penta-acetic acid (DTPA)-renografi;

3. effekten af probiotiske bakterier på det inflammatoriske respons ved endotoxinæmi;

4. effekten af probiotiske bakterier på graden af insulinresistens, bestemt ved euglykæmisk insulinclamping og stabil isotop-teknik;

5. mikropopulationens sammensætning i fæces primært bestemt ved dyrkningsfri molekylærbiologiske metoder; samt

6. effekten af probiotiske bakterier på mikropopulationens sammensætning i fæces primært bestemt ved dyrkningsfri molekylærbiologiske metoder.

7. om en evt. effekt af probiotiske bakterier på mirkopopulationens sammensætning i fæces vedvarer efter indtagets ophør.

**Baggrund**

*Diabetes mellitus type 2*

Diabetes mellitus type 2 (type 2 diabetes) er karakteriseret ved øget insulinresistens i de perifere væv, abnorm insulinsekretion fra beta-cellerne i pancreas og en excessiv glucosefrisætning i leveren, resulterende i hyperglykæmi. Plasmalipider er ofte forhøjede. Incidensen af type 2 diabetes er stigende, også i U-landene, og diabetes vil på verdensplan inden for en overskuelig fremtid formentlig være blandt de førende årsager til morbiditet og mortalitet ^1^.

Den metaboliske dysregulering associeret med type 2 diabetes kan medføre sekundære patofysiologiske forandringer i et eller flere organsystemer med påvirket organfunktion til følge, og type 2 diabetikere har generelt en øget mortalitet og morbiditet sammenlignet med baggrundsbefolkningen. Ca. 10% udvikler kronisk nefropati, og type 2 diabetikere har også, selv uden manifest kronisk nyreinsufficiens, højere risiko end ikke-diabetikere for at udvikle akut nyresvigt i forbindelse med indlæggelseskrævende sygdom ^2^.

Talrige studier har vist en association mellem type 2 diabetes og kronisk forhøjede koncentrationer af cytokiner og andre inflammatoriske markører i plasma ^3^, bl.a. tumornekrotiserende faktor (TNF)-alfa og interleukin(IL)-6. Fra fedt- og muskelvæv frigøres en række signalstoffer, der direkte eller indirekte influerer på insulinfølsomheden i de perifere væv. IL-6, TNF-alfa, adiponectin, leptin, peroxisome proliferator-aktiveret receptor(PPAR)-gamma, PPAR-gamma coactivator(PGC)-1 og plasminogen aktivator inhibitor(PAI)-1 indgår i reguleringen af metabolismen og/eller det inflammatoriske respons, og ændringer i systemiske plasmakoncentrationer af disse stoffer er associeret til insulinresistens og følgevirkninger hertil.

Vi ønsker at undersøge, hvorledes transkriptionen af gener i muskel- og fedtvæv kodende for disse signalstoffer påvirkes hos type 2 diabetikere og raske forsøgspersoner i forbindelse med et standardiseret inflammatorisk respons, udløst ved intravenøs endotoxininjektion (se nedenfor). Herved håber vi afdække sammenhænge mellem inflammation og insulinresistens.

*Sepsis*

Sepsis er defineret som det systemiske inflammatoriske respons på infektion. Ved Gram-negativ sepsis er en væsentlig del af det inflammatoriske respons betinget af frigivelse af lipopolysaccharid (LPS, endotoxin) fra bakteriernes cellevæg. Responset på endotoxin består i frigørelsen af en række pro- og antiinflammatoriske mediatorer, herunder TNF-alfa, IL-1 og IL-6 samt aktivering af en række interrelaterede kaskader, herunder NO-syntese, arachidonsyre-, komplement- og koagulationskaskaden ^4^.

Forekomsten af sepsis er stigende på verdensplan ^5^, og sepsis er den hyppigste dødsårsag på intensivafdelinger. Det skønnes, at der i USA årligt dør 200.000 mennesker som følge af sepsis, svarende til 10 % af alle dødsfald ^6^. Overføres dette tal til danske forhold, svarer det til 5-6000 dødsfald om året som følge af sepsis.

Sepsis medfører ofte dysfunktion af et eller flere organsystemer, f.eks. nyrer, hjerte/kredsløb, koagulationssystem og lunger. Af sepsis-dødsfald skyldes 10% hjertedysfunktion, 40% andre typer cirkulatorisk dysfunktion og 50% multiorgan-dysfunktionssyndrom (MODS), dvs. dysfunktion af mere end ét organsystem ^7^. Mortaliteten ved sepsis stiger jo flere organsystemer, der svigter og med sværhedsgraden af organdysfunktionen ^8^. Patogenesen bag organdysfunktionen antages i hvert fald delvist at være betinget af perfusionsforstyrrelser efterfulgt af vævshypoxi og celledød. Perfusionsforstyrrelserne ses typisk i mikrocirkulationen ^9^. Endothelet spiller formentlig en væsentlig rolle i de patofysiologiske forandringer ved sepsis, idet det dels udtrykker markører som vascular intercellular adhesion molecule (VCAM) og intercellular adhesion molecule (ICAM), der medierer adhæsion og penetration af leukocytter fra karbane til væv, dels ændrer status fra at være anti- til overvejende prokoagulant med nedsat ekspression af trombomodulin og øget ekspression af *tissue factor*, hvilket antages at kunne føre til dannelse af mikrotromboser.

Der er god evidens for en sammenhæng mellem inflammation og insulinresistens. Således har type 2 diabetikere øget risiko for at pådrage sig bakterielle infektioner og sepsis ^10^, og omvendt er sepsis hyppigt associeret med hyperglykæmi og insulinresistens ^11,12^ – både hos diabetikere og ikke-diabetikere. Mortaliteten ved sepsis er endvidere positivt korreleret til graden af insulinresistens. Dette er formentlig udtryk for en sammenhæng mellem sværhedsgraden af det inflammatoriske respons og graden af insulinresistens; alternativt kan der være tale om en selvstændig effekt af insulinresistens og / eller hyperglykæmi på de patofysiologiske manifestationer.

Vi ønsker at undersøge det inflammatoriske respons i relation til patofysiologiske forandringer ved tidlig sepsis. Dette er vanskeligt at opnå ved undersøgelser på patienter med sepsis, dels fordi det nøjagtige starttidspunkt for patienternes sepsis er ukendt, dels fordi der ofte er komplicerende organpåvirkning og primære infektionsfoci. Dyreeksperimentelle studier er en mulighed, men fortolkningen af disse studier vanskeliggøres af, at dyr er relativt resistente over for infektion, således at der skal benyttes doser, der er mange størrelsesordener højere end hos mennesker for at fremkalde tilsvarende klinisk sygdom, og dels fordi forskellige dyremodeller ofte giver helt modstridende data. Den humane endotoxinæmi-model muliggør mekanistiske studier hos mennesker af tidlige fænomener ved sepsis. Selv om modellen som beskrevet nedenfor baserer sig på Gram-negative bakteriebestanddele, og selv om det inflammatoriske respons ved ikke-Gram-negativ sepsis medieres af andre bakteriebestanddele end endotoxin (fx lipoteichonsyre i den Gram-positive bakterievæg), kan signaltransduktionsmekanismer og patofysiologiske forandringer antages at være i det væsentlige ens ved Gram-negativ og ikke-Gram-negativ sepsis.

*Human endotoxinæmi*

Den humane endotoxinæmi-model har til formål at belyse tidlige patogenetiske og patofysiologiske mekanismer ved sepsis. Ved denne model indgives en veldefineret dosis af et ikke-infektiøst reference-endotoxin (Gram-negativ bakteriebestanddel) intravenøst til raske frivillige; Herved udløses en overordentlig reproducerbar og uniform, fuldt reversibel reaktion af timers varighed med kliniske og parakliniske fund, der afhænger af den indgivne dosis. Således ses efter 2-4 ng/kg en influenzalignende tilstand med kulderystelser, hovedpine, myalgier, almen utilpashed og evt. kvalme og opkastning ^13^,og med 500 til 1000 fold stigning i TNF og IL-6, mens indgift af fx 0,3 ng/kg medfører et langt mildere klinisk forløb evt. med lette symptomer i form af kulderystelser, hovedpine, kvalme og temperaturstigning (Erikstrup C, personlig kommunikation, Taudorff S, personlig kommunikation). Der ses 20-60 fold stigning i TNF-alfa og IL-6 (Erikstrup C, upubliserede data), hvilket svarer til et cytokinniveau, der kan måles hos septiske patienter. Både det kliniske og parakliniske forløb af human endotoxinæmi er beskrevet i talrige publikationer på verdensplan siden 1985. Alvorlige bivirkninger er aldrig beskrevet. Vort eget laboratorium har ligeledes stor erfaring med modellen ved indgift af både lave og høje doser endotoxin til raske forsøgspersoner ^14-24^ og har ej heller fundet alvorlige bivirkninger.

*Akut nyresvigt ved sepsis*

Risikoen for akut nyresvigt (*acute renal failure*, ARF) i forbindelse med sepsis er høj. Ved moderat sepsis ses ARF hos ca. 19%, ved svær sepsis ca. 23% og ved septisk shock med samtidig forekomst af positiv bloddyrkning ses ARF i ca. 51% af tilfældene ^25^. ARF har prognostisk betydning ved sepsis, idet mortaliteten hos patienter med både sepsis og ARF er ca. 70% ^26^ og dermed højere end for patienter med enten sepsis (ca. 20%) ^27^ eller ARF alene (ca. 45%) ^28^. Både Gram-positiv og Gram-negativ sepsis medfører risiko for at udvikle ARF. Type 2 diabetikere, både med og uden kronisk nyrepåvirkning, er i højere risiko end ikke-diabetiske patienter for at udvikle akut nyresvigt ^29^, f.eks. i forbindelse med sepsis.

Patogenesen for ARF i forbindelse med sepsis er stadig delvist uafklaret ^30^. Ved Gram-negativ sepsis interagerer endotoxin fra bakteriernes cellevæg med monocytter/makrofager, endothelceller, neutrofilocytter og mesangialceller. Herved frigøres inflammatoriske cytokiner, og koagulations/fibrinolyse-kaskaden aktiveres. Renale endothel- og tubulusceller opregulerer VCAM og ICAM foruden selektiner, cytokiner, kemokiner m.m. Dette medfører leukocytinfiltrering og er medvirkende til initieringen af et inflammatorisk respons i nyren. Både systemisk og lokalt i nyren frigøres som følge af endotoxinpåvirkning en række forskellige vasoaktive mediatorer, herunder platelet activating factor (PAF) ^31^, endothelin-1 (ET-1) ^32^ samt thromboxan A_2_ (TxA_2_) ^33^. Stofferne har en karkontraherende effekt på den afferente og efferente arteriole i nyren, og den øgede koncentration af disse mediatorer under Gram-negativ sepsis kan således antages at påvirke nyreperfusionen og GFR i negativ retning.

Samlet set er udviklingen af ARF i forbindelse med Gram-negativ sepsis formentlig betinget af sammenfaldet mellem de lokale hæmodynamiske følger af endotoxinfrigørelsen med frigørelse af vasoaktive mediatorer, det inflammatoriske respons samt de renale konsekvenser til dén systemiske aktivering af neurohumerale systemer, sympaticus og renin-angiotensin systemet, der finder sted i forbindelse med det endotoxin-udløste fald i systemisk vaskulær resistens. Som anført ovenfor udløses de patofysiologiske processer ved ikke-Gram-negativ sepsis af andre bakteriebestanddele end endotoxin, men der ses aktivering af de samme kaskader som ved Gram-negativ sepsis. En belysning af de patofysiologiske mekanismer ved Gram-negativ sepsis kan derfor antages at have relevans for alle typer sepsis.

Der findes så vidt vi ved kun 3 studier, der har undersøgt forandringer i den renale hæmodynamik på septiske patienter ^34-36^. I disse 3 studier fandt man ved hjælp af PAH-clearance, nyrevenekatetre og termodilutionsteknikker generelt et øget renalt plasma flow (RPF) og en nedsat glomerulær filtrationstrate (GFR). I disse studier er det imidlertid svært at afgøre, hvor patienterne tidsmæssigt befinder sig i det septiske forløb, og dette vanskeliggør anvendelsen af resultaterne til en patogenetisk forståelse af mekanismerne bag nyresvigt. Der er foretaget en del studier af renal hæmodynamik under eksperimentelt fremkaldt sepsis hos dyr. Det eksperimentelle design og de opnåede resultater i disse studier er divergerende ^37^, og resultaterne er derfor vanskelige at overføre til humane forhold. Der findes, så vidt vi ved, ingen publicerede studier, der belyser evt. ændringer i GFR og RPF under eksperimentel human endotoxinæmi. Ved at studere den renale hæmodynamik under eksperimentelt fremkaldt human endotoxinæmi ønsker vi at udnytte, at modellen giver et præcist tidspunkt for påbegyndelsen af det inflammatoriske respons og dermed mulighed for indblik i evt. forandringer under den helt tidlige fase af sepsis, og uden at målingerne konfunderes af samtidige forandringer i den systemiske hæmodynamik. Som markører for en evt. midlertidig påvirkning af tubulusepithelet under lavdosis endotoxinæmi vil vi endvidere undersøge urin og serum for cystatin C samt urin for Na/H exchanger isoform 3. Disse proteiner er blot 2 ud af en række markører, der gennem de sidste år har vist sig at udskilles gennem urinen som udtryk for en evt. påvirkning af tubulusepithelet eller anden nyreskade, enten i forbindelse med sepsis eller som følge af toxisk eller iskæmisk påvirkning ^38^. En øget forekomst af markørproteiner i urinen har formentlig en prognostisk betydning for udviklingen af ARF i forbindelse med sepsis.

*Probiotiske bakterier*

Tarmfloraen spiller en vigtig metabolisk rolle for processer både systemisk og lokalt i tarmen, og dens sammensætning kan påvirkes af kosten. Probiotiske bakterier er levende mikroorganismer af human oprindelse, der indtaget peroralt antages at have en gavnlig effekt på helbreddet ^39^. Indtaget gennem kosten eller som kosttilskud (f.eks. fra surmælksprodukter eller i kapsler) udøver de probiotiske bakterier formentlig deres forskellige sundhedsfremmende egenskaber via modulation af tarmens bakteriesammensætning. Forskellige mælkesyreproducerende bakterier har varierende probiotiske egenskaber med hensyn til at adhærere til tarmepithelceller samt til at hæmme vækst af andre bakterier i tarmen, og visse probiotiske bakterier udøver en immunmodulerende effekt, herunder en forskydning af cytokinprofilen i en mere anti-inflammatorisk retning ^40^.

Lactobaciller udgør en vigtig del af tarmfloraen, og visse stammer menes at have probiotiske egenskaber. Humane studier har vist en gavnlig effekt af *Lactobacillus acidophilus* på forebyggelse og behandling af diaré ^41,42^, bakteriel vaginose ^43-45^, colon irritabilli ^46^ samt en forbedret lactosefordøjelse ^47^. *L. acidophilus* er godkendt til mennesker af FHO/WHO, har i over 25 år været kommercielt tilgængelig som kosttilskud, og findes i surmælksproduktet, bl.a. A-38. *Lactobacillus acidophilus* NCFM er en velkendt stamme og blev første gang isoleret fra en human kilde i 1970’erne. *L. acidophilus* NCFM´s probiotiske egenskaber er undersøgt i mange in vitro og in vivo studier. Således er immunmodulerende egenskaber hos bakteriestammen blevet påvist hos mus ^48^, og man har i humane interventionsforsøg set en ændret sammensætning af lactobaciller i fæces efter indtag af *L. acidophilus* NCFM ^49,50^. I de humane forsøg så man, at sammensætningen af lactobaciller vendte tilbage mod udgangsforholdene efter behandlingsophør, som tegn på at tarmen ikke koloniseredes med *L. acidophilus* NCFM. Genomet er fuldt sekventeret ^51^. I USA er kosttilskuddet *L. acidophilus* NCFM tildelt Safe status (GRAS) ved Food and Drug Administration (FDA). Indtag af lactobaciller opfattes som sikkert uden alvorlige helbredsmæssige risici ^52^.

Type 2 diabetes er associeret med kronisk forhøjet koncentration af cytokiner i plasma. Vi ønsker i dette projekt at undersøge, om indtag af *L. acidophilus* NCFM som kosttilskud - ved at modulere tarmfloraens sammensætning - reducerer det inflammatoriske respons i forbindelse med eksperimentel endotoxinæmi hos både type 2 diabetikere og raske forsøgspersoner. På baggrund af sammenhængen mellem inflammation og insulinresistens, og idet vi antager at *L. acidophilus* NCFM er i stand til at modulere det inflammatoriske respons, vil vi endvidere undersøge, om bakteriestammen påvirker insulinfølsomheden i gunstig retning.

**Hypoteser**

Vi ønsker at teste følgende hypoteser:

1. Efter en intravenøs bolusinjektion af endotoxin ses hos både type 2 diabetikere og raske en midlertidig øget genekspression i fedt- og muskelvæv af cytokinerne IL-1, IL-6, TNF-alfa, PAI-1, leptin og adiponectin, mens ekspressionen af PPAR-gamma og PGC-1 mindskes.
2. Efter endotoxininjektion ses hos både type 2 diabetikere og raske forsøgspersoner en midlertidig reduktion i RPF og GFR . Reduktionen i RPF og GFR er mere udtalt hos diabetikere end hos raske.
3. Efter endotoxininjektion ses midlertidige stigninger i plasma ET-1, plasma PAI-1, plasma- og urin VCAM/ICAM, serum- og urin cystatin C, plasma- og urin PAF og plasma TxA_2_ hos type 2 diabetikere og raske forsøgspersoner. Urinudskillelsen af Na/H exchanger isoform 3 øges i begge grupper.
4. Ændringer i RPF og GFR efter endotoxininjektion er negativt korreleret til ændringer i plasma-VCAM/-ICAM, serum-cystatin C, plasma-PAF samt plasma ET-1, plasma-PAI-1 og plasma TxA_2_.
5. Daglig indtagelse af probiotiske bakterier gennem 4 uger reducerer det inflammatoriske respons efter endotoxininjektion hos både type 2 diabetikere og raske forsøgspersoner.
6. Daglig indtagelse af probiotiske bakterier gennem 4 uger medfører øget insulinfølsomhed påvist ved euglykæmisk insulinclamp og stabil isotop-teknik.
7. Mikropopulationens sammensætning i fæces er forskellig for type 2 diabetikere og raske forsøgspersoner.
8. Indtagelse af probiotiske bakterier påvirker mikropopulationens sammensætning i fæces fra type 2 diabetikere og raske forsøgspersoner.

**Forsøgspersoner.**

I alt inkluderes 24 mænd med type 2 diabetes og 24 raske mænd i alderen 25-80 år. De raske forsøgspersoner aldersmatches type 2 diabetikerne.

*Type 2 diabetikere:*

Inklusionskriterier:

tidligere påvist type 2 diabetes, diagnosticeret i henhold til WHO´s kriterier.

Eksklusionskriterier:

aktuel eller tidligere svær hjerte-, lunge- eller nyresygdom eller anden pågående alvorlig lidelse fraset type 2 diabetes;

behandling med insulin;

dysreguleret hypertension (systolisk blodtryk over 100+ alder, diastolisk blodtryk over 90);

hypertension behandlet med enhver form for angiotensin converting enzyme (ACE)- eller angiotensinreceptorhæmning;

hypertension behandlet med mere end to stofgrupper;

trombocythæmmer- eller antikoagulationsbehandling ud over Hjertemagnyl;

fast peroral antibiotika;

kronisk nyrepåvirkning påvist ved serum-creatinin > 0.150 mM;

systemisk immunsupprimerende behandling fx peroralt steroid.

*Raske forsøgspersoner:*

Eksklusionskriterier:

Indtagelse af lægemidler ud over sæsonbetinget høfebermedicin.

Forsøgspersonerne må ikke have haft infektioner inden for de forudgående 14 dage inden første endotoxinstudie. Hvis de bliver febrile i perioden, hvor der indtages *Lactobacillus acidophilus* NCFM / placebo, udskydes forsøgsdag D og E evt., således at der foreligger en feberfri periode på mindst 14 dage forud for endotoxinstudiet på dag E.

Der vil kun blive udført DTPA-renografi på 16 diabetikere og 16 raske. Dette skyldes logistiske hensyn til patientbelastningen på Klinisk Fysiologisk afdeling, og at vi forventer at kunne påvise en signifikant forskel i RPF for type 2 diabetikere henholdsvis raske ved inklusion af 16 personer fra hver undergruppe (se statistik). Udvælgelsen til DTPA-renografi foregår ved lodtrækning i forbindelse med forundersøgelsen.

12 type 2 diabetikere og 12 raske forsøgspersoner randomiseres til indtagelse af *L. acidophilus* NCFM, mens de øvrige 12 diabetikere og 12 raske udleveres placebo. De udleverede pakningers indhold af enten *L. acidophilus* NCFM eller placebo er blindet for både projektleder og forsøgsperson. Randomiseringen finder sted centralt på KVL ved pakningen af bakteriekultur / placebo.

Forsøgspersonerne rekrutteres ved opslag og annoncering i tidsskrifter, lokalblade og aviser. Interesserede vil kunne kontakte projektleder telefonisk eller pr. E-mail. Såfremt det på baggrund af et kortere telefoninterview vurderes, at personen ikke opfylder eksklusionskriterier, vil han blive inviteret ind til forundersøgelse samt få tilsendt skriftlig forsøgspersoninformation.

**Design og metoder.**

Forsøgspersonerne møder til en forundersøgelse samt til undersøgelser og interventioner på fem efterfølgende projektdage (dag A til E), i alt seks undersøgelsesdage, dog således at forsøgsdag A ikke udføres hos alle forsøgspersoner (se nedenfor). Efter dag A til C, men inden dag D og E, indtager forsøgspersonerne enten *L. acidophilus* NCFM eller placebo i fire uger. Dog vil evt. febril sygdom inden for denne periode kunne medføre en udvidelse af behandlingsperioden, så det sikres, at dag E (endotoxinstudie) udføres mindst 14 dage efter ophør af feber. Dag B og C samt dag D og E udføres over to på hinanden følgende dage.

*Forsøgsdage:*

*L. acidophilus* NCFM / placebo

DTPA-renografi

Muskel / fedt-biopsier samt insulin clamp med stabil isotop teknik

Endotoxininjektion,DTPA-renografi, muskel / fedt-biopsier

Insulin clamp med stabil isotop teknik

Endotoxin-injektion

Dag A

Dag B

Dag C

Dag D

Dag E

2 på hinanden flg. dage

4 ugers mellemrum

2 på hinaden flg. dage

Der udføres således følgende undersøgelser og interventioner:

*- Muskel- og fedtbiopsitagning:*

Alle forsøgspersoner (dvs. 24 diabetikere og 24 raske forsøgspersoner) får taget muskel- og fedtvævsbiopsi i alt 4 gange fordelt på 2 dage ved flg. tilstande:

1. Baseline-undersøgelse (dag B)

2. En time efter injektion af endotoxin (dag C)

3. Tre timer efter injektion af endotoxin (dag C)

4. Syv timer efter injektion af endotoxin (dag C)

*- Insulinclamp:*

Alle forsøgspersoner får udført i alt to insulin-clamps med stabil isotop-teknik ved flg. tilstande:

1. Baseline-undersøgelse (dag B).
2. Efter fire ugers indtagelse af *L. acidophilus* NCFM / placebo (dag D).

*- DTPA-renografi:*

På 16 diabetikere og 16 raske forsøgspersoner foretages i alt tre DTPA-renografier ved flg. tilstande:

1. Baseline-undersøgelse (dag A)

1. 1½ time efter injektion af endotoxin eller placebo (dag C).
2. Seks timer efter injektion af endotoxin eller placebo (dag C)*.*

*- Endotoxininjektion:*

Til alle forsøgspersoner indgives endotoxin to gange på flg. tider:

1. ”Baseline”-undersøgelse, (dag C).

2. Efter 4 ugers indtagelse af *L. acidophilus* NCFM / placebo (dag E).

Forsøgsdagene forløber således:

*Forundersøgelse.*

Mindst to dage forud for 1. forsøgsdag (dag A) optages journal inkl. anamnese og objektiv undersøgelse, EKG og blodtryksmåling. Ved blodprøver screenes for infektionssygdomme, bindevævssygdomme, stofskiftesygdomme, nyresygdomme, og leversygdomme. In- og eksklusion i henhold til ovenfor specificerede kriterier. Forsøgspersonerne informeres mundtligt og skriftligt om projektet. Forsøgspersonen instrueres i at pausere hjertemagnyl dagen før forsøgsdag B og genoptage denne umiddelbart efter forsøgsdag C. Desuden skal antidiabetika og statiner pauseres i 1 uge før insulin-clamps, dvs. forud dag B og D. Præparaterne kan genoptages umiddelbart efter forsøgene på dag C og E. Forsøgspersonerne medgives information om opsamling af fæcesprøver.

Der trækkes lod om evt. udførelse af DTPA-renografi.

*Forsøgsdag A* – DTPA-renografi.

Forsøgspersonen møder på Klinisk fysiologisk afdeling med henblik på udførelse af baseline DTPA-renografi. DTPA udskilles udelukkende ved glomerulær filtration i nyrerne. Efter indgift af radioaktivt mærket DTPA (99mTc-DTPA) er hastigheden, hvormed aktiviteten optages i nyrerne (optagefasen) derfor udtryk for GFR. Ved yderligere beregninger kan det renale plasmaflow også estimeres.

Forsøgspersonen placeres under et gammakamera, hvorefter der indgives en iv bolus injektion af 3 MBq/kg kropsvægt 99mTc-DTPA. Dette svarer til en stråledosis på 1,4 mSv per DTPA-renografi, og derfor en samlet stråledosis på godt 4 mSv for alle renografierne. Ved hjælp af gammakameraet laves dynamiske optagelser i de efterfølgende 21 min. Ved efterfølgende databehandling optegnes renogrammer og beregnes estimeret GFR, RPF samt afløbsparametre for hver nyre. Patientes dirurese noteres ligeledes.

Forsøgspersonen skal drikke mindst 750 ml vand en time inden undersøgelsen, eller hydreres tilsvarende på anden vis.

Forsøgspersonen hjemsendes umiddelbart efter DTPA-renografien.

*Forsøgsdag B* – Baseline muskel- og fedtvævsbiopsi, euglykæmisk insulin-clamp og stabil glucose- isotop teknik.

Forsøgspersonen møder fastende kl. 7 på Center for Inflammation og Metabolisme, afsnit 7641.

Der udtages muskel- og fedtvævsbiopsi. Muskelbiopsier udtages fra vastus lateralis musculus quadriceps femoris efter anlæggelse af lokalbedøvelse (5% lidokain) i hud og underliggende strukturer ned til fascien. Huden incideres med skalpel, og nålebiopsi udtages. Sårrandene samles stramt med plaster. Fedtbiopsierne udtages fra det subcutane væv på abdomen under samme procedure.

Der anlægges herefter 3 venflons, ét i hver cubitalvene og ét i en håndrygsvene. Denne hånd skal være placeret i en varmepude under hele forsøget for at opnå udtag af arterialiseret blod.

I albuevenekateteret indgives fra kl. 8 (t = 0) kontinuerlig infusion af stabilt ^2^[H_2_]-mærket glucose (primingdosis 16 mikromol/kg, infusionshastighed 0,22 mikromol/kg/min). Infusionshastigheden nedsættes til 50% af udgangshastigheden efter 2½ time.

Kl. 10.30 (t = 150 min) opstartes 2½ timers euglykæmisk insulin-clamp. Hurtigtvirkende insulin (Actrapid®, Novo Nordisk, Danmark) infunderes kontinuerligt med en hastighed på 0,08 IE/min/m2. Glucose 20% infunderes via en computer-kontrolleret infusionspumpe med hastigheder indstillet til at fastholde et blod-glucose på fasteniveau 5,0 mM. For kontinuerligt at monitorere sukker og kaliumkoncentrationen i blodet, tages der under clampen blodprøver fra venflonet i håndryggen, initialt hver 5. minut, og efter den første time hver 10. minut. Kaliumkoncentrationen fastholdes på baselineniveau, ved kontinuerligt at indgive isotonisk natriumklorid med kalium (51 mM).

Der tages blodprøver til tracerbestemmelse før opstart af glucose isotop infusionen (t = 0) og til tiderne t = 120, 130, 140, 150, 250, 260, 270 og 300 minutter.

Forsøgspersonen hjemsendes ca. 1 time efter clampens afslutning, når blodsukkeret ikke udviser faldende tendens.

*Forsøgsdag C* – Endotoxin-indgift, DTPA-renografi samt muskel- og fedtvævsbiopsitagning.

Forsøgsdag C er placeret dagen efter forsøgsdag B og inden start af indtagelse af probiotiske bakterier / placebo. Forsøgspersonen møder fastende på Intensiv afd. 4131 kl. 7. Der anlægges venflons i to overekstremitetsvener til henholdsvis blodprøvetagning og væske / endotoxinindgift. Monitorering med kontinuerlig måling af rektaltemperatur, puls, iltsaturation, non-invasiv måling af hjertets minutvolumen med finometer og EKG. Blodtryk registreres hver 15. minut. Der måles timediureser ved at opsamle urin i kolbe hver time i 8 timer.

Kl. 8 indgives endotoxin som bolus, 0,3 ng/kg i.v. Herefter overvåges forsøgspersonen til stadighed af en læge, der har dette som eneste opgave.

Kl. 9.30 og kl. 14 foretages på udvalgte forsøgspersoner DTPA-renografi på Klinisk fysiologisk afdeling under lægeledsagelse.

Kl. 9, kl. 11 og kl. 15 udtages muskel- og fedtvævsbiopsier med samme teknik som på dag B.

Der tages blodprøver fra venflon umiddelbart inden indgift af endotoxin (0 timer) samt til tiderne 1, 2, 3, 4, 5, 6, 7 og 8 timer. Urinprøver fra den opsamlede urin med henblik på urinanalyse tages på samme tidspunkter.

Forsøgspersonen hjemsendes tidligst 8 timer efter endotoxinindgiften og skal da være subjektivt velbefindende med tp. < 38°C.

*Forsøgsdag D* – Euglykæmisk insulin-clamp inkl. stabil glucose isotop teknik

Forsøgspersonen møder fastende kl. 7 på Center for Inflammation og Metabolisme, afsnit 7641, med henblik på udførelse af euglykæmisk insulin-clamp og stabil glucose isotop teknik svarende til dag B; dog udtages ikke biopsier.

*Forsøgsdag E*- Endotoxin-indgift.

Forsøgsdag E placeres dagen efter forsøgsdag D og mindst 14 dage efter en evt. febril tilstand eller infektion under indtagelse af probiotiske bakterier / placebo. Forsøgspersonen møder fastende kl. 7 på Intensiv afdeling 4131 Der anlægges 2 venekatetre i overekstremitetsvener til henholdsvis blodprøvetagning samt væske / endotoxin-indgift. Monitorering med kontinuerlig rektaltemperatur-måling, puls, iltsaturation og EKG samt blodtryk hver 15. minut.

Kl. 8 indgives endotoxin som intravenøs bolus, 0,3 ng/kg. Herefter overvåges forsøgspersonen til stadighed af en læge, der har dette som eneste opgave.

Der tages blodprøver fra venekateter umiddelbart inden indgift af endotoxin (0 timer) samt til tiderne 1, 2, 3, 4, 5, 6, 7, 8 timer. Forsøgspersonen hjemsendes tidligst 8 timer efter endotoxin-injektion og skal da være subjektivt velbefindende med tp. < 38°C.

*Indgift af probiotiske bakterier* – tidsrummet mellem forsøgsdag C og forsøgsdag D.

12 type 2 diabetikere og 12 raske forsøgspersoner er randomiseret til at indtage 1 g *L. acidophilus* NCFM 2 gange dagligt i en periode på 4 uger, mens de øvrige 12 diabetikere og 12 raske i samme periode indtager placebo. Forsøgspersonen må ikke indtage andre surmælksprodukter i denne periode, men skal i øvrigt tilstræbe at spise så tæt på sin vanlig kost som muligt. Indtaget opstartes om aftenen på forsøgsdag C og afsluttes 4 uger senere, sv.t. om morgenen på forsøgsdag D.

### Fæcesprøver

Forsøgspersonen skal i alt opsamle og indsende til KVL fire fæcesprøver. Fæcesprøverne tages 1) 7 dage før indtaget af *L. acidophilus* NCFM påbegyndes, 2) mindre end 24 timer forud for dag B, 3) mindre end 24 timer for indtaget af *L. acidophilus* NCFM afsluttes dag D, og 4) 7 dage efter dag D. Vedledning i udtagelse og opbevaring af fæcesprøver gives dels mundtligt, dels via en særskilt skriftlig vejledning fra KVL ved forundersøgelsen. Prøverne sendes friske til KVL, der er ansvarlig for den efterfølgende opbevaring, håndtering og analysering af prøverne.

**Analyser**

Der foretages følgende analyser:

*Forundersøgelse:*

*Blod:* Hæmoglobin, trombocytter, koagulationsfaktor II, VII, X, leukocytter, CRP, kreatinin, carbamid, ASAT/ALAT, blodglucose, TSH, cholesteroler og triglycerider.

*Forsøgsdag B og C – Muskel- og fedtvævsbiopsitagning:*

*Muskel og fedtvæv:* mRNA for IL-1, IL-6, TNF-alfa, PGC-1, PPAR-gamma, leptin, adiponectin, PAI-1.

*Forsøgsdag B og D – insulin-clamp:*

*Blod:* ^2^[H_2_]-glucoseisotop, blod-glucose, kalium.

*Forsøgsdag C og E - endotoxinindgift:*

*Blod (fuldblod / serum / plasma):*

EDTA-plasma og serum fra vene til bestemmelse af TNF-alfa, IL-1, IL-6.

Almindelige klinisk-biokemiske analyser: hæmoglobin, elektrolytter, leukocyttal og differentialtælling, trombocytter, C-reaktivt protein, koagulationsfaktor II+VII+X, kreatinin, carbamid, TSH, triglycerider, cholesteroler og B-glucose.

Veneplasma til bestemmelse af ET-1, VCAM og ICAM, PAF og TxA2 (*kun forsøgsdag C)*

Vene-serum til bestemmelse af cystatin C *(kun forsøgsdag C)*

Veneplasma til bestemmelse af renin og ANG-II *(kun forsøgsdag C)*

*Urin (kun forsøgsdag C):*

VCAM, ICAM, PAF, urin Na/H exchanger isoform 3, cystatin C samt albumin.

Flg. analysemetoder benyttes:

Cytokiner: Plasmakoncentrationer af TNF-alfa, IL-1 og IL-6 bestemmes ved ELISA.

ET-1: Plasmakoncentrationer bestemmes ved radioimmunoassay.

TxA2: Plasmakoncentrationer bestemmes på speciallaboratorium.

Renin: analyseres på speciallaboratorium

Angiotensin II: analyseres på speciallaboratorium

VCAM, ICAM, Cystatin C, PAF i urin og blod: analyseres på speciallaboratorium

Urin Na/H exchange isoform 3 i urin: analyseres på speciallaboratorium

PGC-1, PPAR, leptin, adiponectin, PAI-1, IL-1, IL-6 og TNF-alfa mRNA monitoreres med revers transkriptase(RT)-polymerase kædereaktion(PCR). For visse markører vil proteinexpressionen blive evalueret ved immunohistokemi og Western Blot.

##### Materialer

*Endotoxin:* Der anvendes et standard reference *E. coli* endotoxin (lot EC-6) (US Bureau of Biologica, Food and Drug Administration, Bethesda, Maryland, USA). Dette endotoxin har været anvendt i nærværende forskergruppers tidligere delprojekter vedrørende human endotoxinæmi.

*Probiotiske bakterier:* Der anvendes den probiotiske bakteriestamme *Lactobacillus acidophilus* NCFM. Der foreligger aftale mellem KVL og Danisco om levering af den frysetørrede bakteriekultur og placebo. Små pakninger indeholdende 1 g bakterier/placebo sendes fra KVL til Center for Inflammation og Metabolisme, som vil stå for udlevereingen af pakningerne til forsøgspersonerne. Indholdet af pakningerne er blindet for projektleder og forsøgsperson; randomiseringskoden findes på KVL. Inden udlevering opbevares bakterierne ved –80^o^ og har da praktisk taget ubegrænset holdbarhed. Efter udlevering til forsøgspersonerne skal bakteriekulturen opbevares i køleskab indtil indtagelse. 1 gram frysetørret bakteriekultur indeholder 10^10^ levende bakterier. Til sammenligning indeholder 1 gram A-38 10^8^ levende *L. acidophilus*, dvs. ved at indtage 100 g A-38 fås samme mængde bakterier som 1 projektpakning med *L. acidophilus* NCFM. Den frysetørrede kultur/placebo indtages 2 gange dagligt blandet i et afkølet måltid.

Placebo består af de hjælpestoffer, der ligeledes indgår i pakningerne med *L. acidophilus* NCFM, og svarer til de hjælpestoffer, der indgår kommercielt tilgængelige produkter, f.eks. A-38.

# **Statistik**

Der anvendes parametrisk statistik, hvis parametrene viser sig at være normalfordelt. P-værdi mindre end 0,05 anses for at være statistisk signifikant.

*Styrkeberegning*:

Ad inflammatorisk respons ved endotoxinæmi: tidligere studier i laboratoriet har godtgjort en 20 til 60 fold stigning i plasmacytokinkoncentrationer efter lavdosis endotoxininjektion 0,3 ng/kg (Erikstrup et al., upublicerede data). Denne stigning vil være signifikant ved inklusion af ganske få forsøgspersoner (tre forsøgspersoner ved anvendelse af parret t-test). Inklusion af 24 forsøgspersoner i hver gruppe anses derfor for fuldt tilstrækkelig til at påvise en stigning i plasmacytokinkoncentration efter endotoxininjektion analyseret ved parret t-test.

Ad renal hæmodynamik under endotoxinæmi: Nulhypotesen er, at der ikke ses en ændring i RPF under endotoxinæmi sammenlignet med baseline-målingen. Under antagelse af en intra-individuel variationskoefficient i nyreflow på 20%, 2α = 0,05, 1-β = 0,8 og minimal relevant difference på 20%, skal der inkluderes 16 personer i den parrede sammenligning af RPF før og under endotoxæmi. Samtidigt vil det være muligt at detektere en 30% ændring i RPF på med 2α = 0,01 og 1-β = 0,9.

Ad insulinresistens, raske vs. diabetikere: Dette er en uparret sammenligning. Under antagelse af en glucoseinfusionsrate (GIR) for raske forsøgspersoner på 7+/-1 (mean+/-SD) mg/kg/min, samme SD hos type 2 diabetikere, N=24 i begge grupper, 2α=0,05 og 1-β=80%, vil det med en uparret t-test være muligt at detektere en forskel ved baseline i GIR på 2,8 mg/kg/min (=40% af mean GIR) mellem grupperne.

Ad insulinresistens, effekt af probiotisk behandling vs. placebo: Under ovenstående antagelser vil det ved uparret sammenligning mellem forsøgspersoner behandlet med probiotiske bakterier og forsøgspersoner behandlet med placebo være muligt at detektere samme forskel (GIR 2,8 mg/kg/min) med et N=24 i begge grupper. For diabetikere hhv. raske forsøgspersoner hver for sig er der tale om et N=12 i begge grupper, og den detekterbare difference bliver herefter 4,0 mg/kg/min (dvs. for sammenligning i GIR for diabetikere behandlet med probiotiske bakterier vs. diabetikere behandlet med placebo). Denne sammenligning suppleres med en mere følsom parret sammenligning, se nedenfor.

Ad insulinresistens, intraindividuel effekt af probiotisk behandling / placebo: Dette er parrede sammenligninger både hos raske og type 2 diabetikere. Nødvendig N for at detektere en forskel ved en parret test er minimum 3, og et N=12 anses derfor for tilstrækkeligt til dette formål.

Ad effekt af probiotiske bakterier / placebo på inflammatorisk respons: Dette er ligeledes en parret sammenligning, og der gælder samme betingelser som angivet ovenfor.

**Risici, bivirkninger og ubehag.**

*Endotoxin*

Vores hidtidige erfaringer med symptomerne ved intravenøs endotoxininjektion hos raske forsøgspersoner er i overensstemmelse med beskrivelser i litteraturen. Den anvendte dosis, 0,3 ng/kg, er relativt lav, men hos nogle forsøgspersoner kan forekomme influenzalignende symptomer i form af hovedpine, kulderystelser, temperaturstigning, pulsstigning, muskelsmerter og/eller kvalme. Der forventes ingen påvirkning af blodtryk.

Endotoxin er ikke tidligere givet i eksperimentel sammenhæng til type 2 diabetikere. Vi forventer imidlertid ikke, at det kliniske respons adskiller sig væsentligt fra det, der ses hos raske forsøgspersoner.

## *Kateterisering*

Der anlægges venflons mhp. indgift af endotoxin, den stabile glucoseisotop og væske samt til blodprøvetagning.

### Isotoper & strålebelastning

Der anvendes en radioaktiv isotop (^99^mTc) til udførelse af DTPA-renografien. Ved én renografi indgives radioaktivitet i en mængde, der medfører en stråledosis på 1,4 mSv. De 3 planlagte renografier giver således en strålemængde på godt 4 mSv, svarende til ca. to gange den årlige baggrundsstråling. Denne strålemængde vurderes at øge risikoen for at dø af kræft med 0,02 procentpoint, dvs. fra 25% til 25,02 %. Der er ingen smerte forbundet med at få udført renografi.

Den stabile glucose-isotop, der anvendes i forbindelse med insulin-clampen medfører ingen radioaktiv belastning.

*Muskel- og fedtbiopsier*

Der tages i alt 4 muskel- og 4 fedtvævsbiopsier svarende til projektdag B og C. Der anlægges lokalbedøvelse inden biopsitagning, hvilket mindsker ubehaget. Ved udtagelse af muskelvæv der dog ofte på trods af lokalbedøvelsen føles et sug eller en trykkende fornemmelse, sjældent en jagende smerte. Der kan sjældent opstå en blodansamling og yderst sjældent en mindre føleforstyrrelse, som forsvinder af sig selv igen. Der kan i mindre grad forekomme smerter og ømhed ved udtagning af fedtbiopsien.

*Blodprøver*

Den samlede udtagne blodmængde ved forundersøgelsen samt forsøgsdagene B og C overstiger ikke 300 ml. Ved forsøgsdagene D og E 4 uger senere udtages yderligere maximalt 300 ml blod.

### Probiotiske bakterier

*L. acidophilus* NCFM er et almindeligt anvendt kosttilskud. Der er ikke rapporteret om bivirkninger, risici eller ubehag ved indtag af *L. acidophilus* NCFM i de i projektet anvendte mængder.

**Forsikringsforhold**

Forsøgspersonerne er under forsøget omfattet af Rigshospitalets patientforsikring.

**Sted for udførelse af forsøget og projektets gennemførlighed**

Rigshospitalet, Center for Inflammation og Metabolisme afsnit 7641, Intensiv Terapi Afsnit 4131 samt Klinisk Fysiologisk og Nuklearmedicinsk Afdeling.

Projektet er et samarbejde mellem følgende afdelinger på Rigshospitalet: Intensiv Terapi Afsnit 4131, Abdominalcentret; Klinisk Fysiologisk og Nuklarmedicinsk Afdeling, Diagnostisk Center, Epidemiafdeling M (Center for Inflammation og Metabolisme), Finsencentret, Nefrologisk Afdeling P, Abdominalcentret. KVL deltager i den del af projektet, der hidrører *L. acidophilus* NCFM ved at udlevere pakninger med bakterierkultur eller placebo til CIM og undersøger de opsamlede fæcesprøver. Deltagerne fra disse afdelinger har erfaring med den kliniske baggrund for projektet såvel som de anvendte metoder. De involverede laboratorier har afgivet tilsagn om deltagelse i projektet og har erfaring og ekspertise med de anvendte metoder. De fornødne materialer er til stede og tilgængelige.

**Forsøgets forventede varighed**

Påbegyndes snarest og forventes afsluttet inden for 12 måneder.

**Honorar**

Der ydes en godtgørelse på 4500 kr. Udføres der DTPA-renografi, modtager forsøgspersonen yderligere kr. 500,-. Godtgørelsen dækker påført ubehag, tabt arbejdsfortjeneste og transportudgifter.

**Støtte til projektet**

Undersøgelsen af sammenhængen mellem insulinresistens, inflammatorisk respons og probiotiske bakteriers evt. helbredsfremmende effekt er finansieret af Det Strategiske Forskningsråd og indgår i et større samarbejde mellem Center for Inflammation og Metabolisme, Rigshospitalet, KVL, DTU og DPU. Projektet planlægges udført som ph.d.-projekt for Sofie Andreasen, hvis løn samt ph.d.-studieafgift er indeholdt i bevillingen fra Det Strategiske Forskningsråd. KVL leverer *L. acidophilus* NCFM samt placebo, som er fremstillet af Danisco. Til udførelse af DTPA-renografi m.h.p. bestemmelse af RPF/GFR i forbindelse med human endotoxinæmi er modtaget et legat på kr. 100.000,- fra Det Medicinske Selskab i København. Der søges om yderligere fondsstøtte fra offentlige og private fonde. Ud over, at Danisco er leverandør af probiotiske bakterier / placebo, er der ingen kommercielle interesser i eller støtte til projektet.

**Publikation**

Der planlægges flere publikationer i engelsksprogede tidsskrifter. Førsteforfatter er Sofie Andreasen, dog er Nadja Larsen førsteforfatter på manuskripter, hvor hovedindholdet er af mikrobiologisk karakter. Forfatterliste og rækkefølge i henhold til Vancouverreglerne. I tvivlstilfælde afgøres forfatterrækkefølgen af Kirsten Møller og Mogens Jakobsen. Danisco har ingen indflydelse på publikationsstrategien.

**Deltagere**

Projektansvarlig: Sofie Andreasen

Klinisk ansvarlig: Bente Klarlund Pedersen er klinisk ansvarlig for den del af projektet, der foregår på Center for Inflammation og Metabolisme, mens Jan Bonde er klinisk ansvarlig for den del, der foregår på intensiv 4131.

Øvrige deltagere: som anført på titelblad.

**Etiske overvejelser**

Endotoxinindgift op til 2 ng/kg hos raske forsøgspersoner er tidligere godkendt af De Videnskabsetiske Komitéer for Københavns og Frederiksberg Kommuner (protokolnr. (KF) 01-144/98). Undersøgelse af nyrernes gennemblødning ved DTPA-renografi efter lav-dosis endotoxinæmi hos raske forsøgspersoner er tidligere godkendt til samme forskergruppe (protokol nummer (KF) 01-296748). Insulin clamping og infusion af stabile glucose isotoper er tidligere godkendt af VEK på raske (KF 01-006/04) og diabetikere (KF 01 – 048/04) og er sikre og velafprøvede metoder. Der er talrige gange tidligere givet tilladelse til fedt- og muskelbiopsier (KF 12-048/03, 11-048/03, 11-112/01, 12-060/01, 02-201/98, 02-010/00). Aktuelle protokol søges nu godkendt med henblik på undersøgelse af RPF og GFR ved hjælp af DTPA-renografi hos type 2 diabetikere under eksperimentel endotoxinæmi og undersøgelse af *L. acidophilus* NCFM´s betydning for insulinresistens og det inflammatoriske respons hos type 2 diabetikere og raske forsøgspersoner.

Dette forsøg udføres på type 2 diabetikere og raske frivillige forsøgspersoner. Diabetikerne har ikke andre alvorlige konkurrerende lidelser. Forsøgspersonerne indgives lavdosis endotoxin, 0,3 ng/kg. Dette kan efter vores erfaring hos nogle raske forsøgspersoner medføre lette influenzalignende symptomer i form af hovedpine, kulderystelser, temperaturstigning, pulsstigning, muskelsmerter og/eller kvalme. Blodtryk er uændret. Paraklinisk registreres en signifikant, men forbigående stigning i pro- og antiinflammatoriske cytokiner. Vi har i vores tidligere forsøg set, at disse forandringer typisk varer 6 timer, men ikke over 24 timer, og der er ej heller i litteraturen rapporteret om blivende mén af nogen art efter eksperimentel human endotoxinæmi. Vi anser derfor endotoxinindgiften for risikofri. Der er ikke tidligere i eksperimentel sammenhæng indgivet endotoxin til type 2 diabetikere. Vi forventer dog ikke, at det kliniske respons vil være markant anderledes hos personer med type 2 diabetes sammenlignet med raske forsøgspersoner. Forsøgspersonerne monitoreres som tidligere forudsat intensivt, både af sikkerhedshensyn samt af hensyn til forsøget.. Der vil til stadighed være en læge til stede, hvis eneste opgave er at overvåge forsøgspersonens kliniske tilstand. Anlæggelse af venflons og den afgivne blodmængde følger standarden for forsøg af denne karakter og anses for at være risikofrit.

Den indgivne mængde radioaktive isotop under de 3 planlagte DTPA-renografier afgiver en samlet strålemængde på godt 4 mSv, svarende til 2 års baggrundsstråling i Danmark. Dette vurderes at øge risikoen for at dø af kræft med 0,02 procentpoint, dvs. fra 25% til 25,02 %. DTPA-renografi er ikke forbundet med ubehag.

Insulin clamping og infusion af stabile glucose isotoper er velgennemprøvede og ufarlige modeller. Blodsukker og kaliumkoncentrationen overvåges tæt under hele clampen. Glucose isotopen er stabil og medfører derfor ingen strålebelastning.

Muskelbiopsitagning kan være forbundet med en vis smerte eller ømhed, hvorimod fedtvævsbiopsitagningen er stort set uden gener. Der kan opstå blødning med efterfølgende hæmatom og følelse af ”trælår”. Der kan i sjældne tilfælde overskæres en perifer nerve med efterfølgende kutane føleforstyrrelser. Følesansen normaliseres over uger til måneder. Biopsierne udtages af erfaren læge. Forskningsgruppen har langvarig erfaring med hensyn til biopsitagning. Der foretages skønsvist 1000 biopsier årligt i Center for Inflammation og Metabolisme.

*L. acidophilus* NCFM har i ca. 25 år været kommercielt tilgængelig som kosttilskud. Den daglige indgift af den i forsøget anvendte *L. acidophilus* NCFM svarer til, hvad der findes af *L. acidophilus* i 200 ml A-38. Placebo består af hjælpestoffer, der findes i både den til forsøgspersonerne udleverede *L. acidophilus* NCFM samt i kommercielt tilgængelig A-38. Indgiften anses for at være risikofri.

Der anvendes kun raske mænd eller mandlige type 2 diabetikere uden konkurrerende lidelser som forsøgspersoner. Betalingen for deltagelse i forsøget anses at stå i rimelig forhold til den forbrugte tid og ubehag for forsøgspersonen.

Dette projekt vil bidrage til en bedre forståelse af forskellige aspekter ved det inflammatoriske respons og dets følgevirkninger. Patienter med insulinresistens og type 2 diabetes har ofte kronisk forhøjet plasma cytokinniveau, og i forbindelse med sepsis vil insulinfølsomheden ofte være påvirket hos raske og yderligere nedsat hos type 2 diabetikere. Projektet søger at afdække mulige mekanismer til associationen mellem inflammation og insulinresistens, idet vi undersøger, hvorledes det inflammatoriske respons under eksperimentel endotoxinæmi hos type 2 diabetikere og raske forsøgspersoner påvirker ekspressionen af metabolismeregulerende gener i muskel- og fedtvæv. Vores håb er, at en bedre forståelse af mekanismerne bag udvikling af insulinresistens kan medvirke til at forebyggelse og behandling af inflammationsassocierede lidelser som sepsis, type 2 diabetes, metabolisk syndrom og fedme optimeres.

Endotoxinmodellen tjener i dette projekt til dels at afdække mulige patogenetiske sammenhænge mellem inflammation og insulinresistens, dels som en eksperimentel sepsismodel. Mange sepsis-patienter udvikler ARF, risikoen herfor er øget hos type 2 diabetikere, men patogenesen er stadig delvist uafklaret og antages at være en kombination af bl.a. inflammatoriske og hæmodynamiske forstyrrelser, både systemisk og renalt. Med projektet ønsker at vi at belyse mulige mekanismer til det akutte nyresvigt ved sepsis. På længere sigt er håbet, at et øget kendskab til de patofysiologiske forhold bag ARF ved tidlig sepsis kan medvirke til, at de profylaktiske og terapeutiske tiltag optimeres, således morbiditet og mortalitet som følge af sepsis nedbringes.

Endelig undersøger vi probiotiske bakteriers betydning for det inflammatoriske respons og for insulinresistens. Vi håber, at påvisning af en evt. antiinflammatorisk og dermed helbredsfremmende effekt på længere sigt kan implementeres i befolkningen.

Vi har i projektet ikke planlagt DTPA-renografi *efter* indgift af *L. acidophilus* NCFM. Dette skyldes, at vi ikke foreventer at se en så kraftig antiinflammatorisk effekt efter 4 ugers indtag, at det skulle have indflydelse på GFR og RPF i forbindelse med endotoxinæmi. Det forekommer derfor ikke rimeligt at udsætte forsøgspersonerne for yderligere strålebelastning i form af endnu en DTPA-renografi. Skulle vores resultater imidlertid vise en så kraftig påvirkning af det inflammatoriske respons ved indgift af *L. acidophilus* NCFM, at en ændret nyrefunktion i forbindelse med endotoxinæmi kan forventes, da indsendes tillægsprotokol med henblik på udførelse af DTPA-renografi før og efter indgift.

Selv om vi ikke forventer en sammenhæng mellem nyreperfusion og *L. acidophilus* NCFM, har vi alligevel valgt både at foretage undersøgelser af den renale hæmodynamik under endotoxinæmi og undersøgelser af de probiotiske bakteriers indflydelse på det inflammatoriske respons og insulinresistens på samme gruppe af forsøgspersoner. Der er i høj grad fælles tilgrundliggende patogenetiske mekanismer til sepsis, nyresvigt, ekspression af metabolismeregulerende gener, akut og kronisk inflammation og diabetes. Disse mekanismer vil vi studere i dette projekt, med kosttilskuddet *L. acidophilus* NCFM som en indlagt intervention. Det er derfor vores opfattelse, at det både fagligt og logistisk er hensigtsmæssigt at gennemføre undersøgelsen som et stort sammenhængende studie, frem for at opdele undersøgelsen i flere mindre projekter.

**Lægmandsprotokol**

Type 2 diabetes, populært kaldet gammelmandssukkersyge, er karakteriseret ved et for højt indhold af sukker og fedtstoffer i blodet. Det forhøjede blodsukker skyldes bl.a. en nedsat følsomhed for og dermed nedsat effekt af insulin i vævene. Diabetespatienter har en kronisk irritationsreaktion (inflammation) i kroppen, som er kendetegnet ved en ændret produktion af en række signalstoffer, der kan påvirke følsomheden for insulin. Det er muligt, at en reduktion i denne inflammationsreaktion kan medføre en bedring i diabetes-tilstanden. Mange diabetikere udvikler på længere sigt, som følge af den kroniske inflammation kombineret med de forhøjede blodsukre og fedtstoffer, nedsat organfunktion i en række organsystemer, herunder i nyrer, øjne, nerver og hjerte/karsystemet.

Sammenhængen mellem kronisk inflammation og nedsat insulinfølsomhed er ikke fuldt afklaret. Vi ønsker derfor at undersøge om en inflammatorisk reaktion, i dette projekt udløst af en eksperimentel bakterieinfektion, påvirker aktiviteten af gener med betydning for insulinfølsomheden i muskler og fedtvæv hos både type 2 diabetikere og raske forsøgspersoner. Den eksperimentelle bakterieinfektion etableres ved indgift i blodet af et bakterieprodukt, *E. coli* endotoxin.

Diabetikere har øget risiko for at pådrage sig bakterieinfektioner. Infektionerne er ofte alvorligere, og dødeligheden i forbindelse med de sværere infektioner er højere. Svære bakterieinfektioner ledsaget af organsvigt (nyrer, hjerte/kredsløb, lever/tarm, hjerne, lunger samt blodets størkningsevne) (sepsis) er den hyppigste dødsårsag blandt patienter, både diabetikere og ikke-diabetikere, på intensiv afdelinger. Ændret blodtilførsel til organerne, ændret organ-stofskifte og produktion af signalstoffer, der påvirker organernes funktion, kan alle tænkes at være medvirkende til udviklingen af organsvigt. Vi vil i dette projekt undersøge, om en eksperimentel bakterieinfektion hos type 2 diabetikere og raske forsøgspersoner midlertidigt påvirker blodtilførslen til nyrerne samt nyrernes filtreringsegenskaber.

Man har i mange år haft en formodning om, at surmælksprodukter med mælkesyrebakterier som kosttilskud har en lang række gavnlige effekter på helbreddet. Flere undersøgelser tyder på at indgift af mælkesyrebakterier kan påvirke vores immunforsvar og muligvis ændre kroniske inflammationstilstande i gunstig retning. Vi vil undersøge, om indtag af mælkesyrebakterien *Lactobacillus acidophilus* NCFM, der allerede er kommercielt tilgængelig i surmælksprodukter, kan påvirke vores immunforsvar således, at sværhedsgraden af en eksperimentelt fremkaldt inflammation mindskes. Med sammenhængen mellem inflammation og nedsat insulinfølsomhed vil vi ligeledes undersøge, om indgift af *Lactobacillus acidophilus* NCFM kan bedre følsomheden for insulin. Endelig vil der blive taget afføringsprøver med henblik på undersøgelse af en evt. ændring i tarmfloraen som følge af indtaget af *Lactobacillus acidophilus* NCFM.

### Forsøgspersoner

Forsøgspersonerne er 24 voksne mandlige type 2 diabetikere uden andre alvorlige lidelser og 24 raske mænd. Forud for forsøget udføres en undersøgelse, hvor der tages EKG (registrering af hjertets elektriske aktivitet) og blodprøver, og forsøgspersonen undersøges for tegn til sygdom og udspørges om symptomer herpå. Ved tegn til alvorligere sygdom (fraset diabetes hos diabetikerne) ekskluderes forsøgspersonen. Forsøgspersonerne fordeles herefter ved lodtrækning til at skulle indtage henholdsvis mælkesyrebakterien *Lactobacillus acidophilus* NCFM (12 diabetikere og 12 raske) eller det uvirksomme placebo (12 diabetikere og 12 raske). Fordelingen er blindet for både forsøgspersoner og forsøgsledere. Ved lodtrækning udvælges blandt de 48 forsøgspersoner endvidere 16 raske og 16 diabetikere til at få foretaget DTPA-renografi (nyrefunktionsundersøgelse, hvor nyrens blodtilførsel og filtreringsegenskaber måles). Dette skyldes, at det af praktiske og statistiske grunde ikke er muligt hhv. nødvendigt at foretage DTPA-renografi på alle de patienter, der indgår i den del af projektet, hvor der undersøges for effekten af *Lactobacillus acidophilus* NCFM.

*Følgende undersøgelser indgår som led i projektet:*

Aktiviteten af forskellige gener i muskler og fedtvæv med betydning for insulinfølsomheden undersøges hos type 2 diabetikere og raske før og umiddelbart efter endotoxinindgift. Hermed belyses inflammationens betydning for insulinfølsomhed.

Nyrens blodtilførsel og nyrens filtreringsegenskaber måles ved hjælp af i alt 3 DTPA-renografier, dels på dag A, dels på dag C 1½ og 6 timer efter endotoxin-indgift.

Mælkesyrebakteriers betydning for sværhedsgraden af betændelsesreaktion i forbindelse med indgift af endotoxin hos type 2 diabetikere og raske forsøgspersoner undersøges.

Mælkesyrebakteriers betydning for følsomheden for insulin hos type 2 diabetikere og raske undersøges ved hjælp af en såkaldt insulinclamp teknik og anvendelse af et sukkersporstof.

Mælkesyrebakteries effekt på tarmfloraens sammensætning i afføring.

*Forsøgets forløb.*

Projektet forløber over en forundersøgelsesdag samt 4 til 5 forsøgsdage; (A), B, C, D,og E.

Dag A møder kun de personer, der skal indgå i undersøgelsen af nyregennemblødningen med henblik på udførelse af DTPA-renografi. Når DTPA-renografien er udført, går forsøgspersonen hjem, hvilket er vanlig praksis også for patienter, som i diagnostisk øjemed får udført denne undersøgelse.

Efter dag A møder alle forsøgspersoner til de efterfølgende forsøgsdage.

Dag B møder forsøgspersonen kl. 7 til en muskel- og fedtvævsprøve samt undersøgelse af insulinfølsomheden og sukkerstofskiftet. I lokalbedøvelse udtages muskelvævsprøver fra en stor muskel på lårets forside. Insulinfølsomheden og sukkerstofskiftet undersøges ved hjælp af en såkaldt euglykæmisk insulin-clamp teknik med samtidig anvendelse af et sukkersporstof. Forsøgspersonerne får anlagt drop i to armvener og et i en håndrygsvene. Der opstartes infusion med sukkersporstoffet, og 2½ time senere startes 2½ times løbende indgift af insulin og sukker med fastholdelse af blodsukkerniveauet på fasteværdier. Der tages hyppige blodprøver til måling af blodsukker og blodsalte.

Dag C møder forsøgspersonen kl. 7. Ved ankomsten til afdelingen anlægges to drop i armvener med henblik på endotoxinindgift, væskeindgift og blodprøvetagning. Der registreres endvidere kontinuerligt temperatur, EKG, puls, hjertets pumpeevne, og blodets iltmætning, mens blodtryk måles hver 15. minut. Kl. 8 indgives endotoxin. Kl. 9, 11 og 15 tages muskel- og fedtvævsprøver. Hos den gruppe af patienter, der skal have foretaget undersøgelse af nyregennemblødningen, foretages endvidere DTPA-renografi kl. 9.30 og kl. 14. Forsøgspersonen forbliver indlagt i mindst 8 timer efter indgift af endotoxin, og indtil personen er velbefindende.

Forsøgsdag D møder forsøgspersonen m.h.p. ny undersøgelse af insulinfølsomhed sv.t. forsøgsdag B. Forsøgsdag E (dagen efter forsøgsdag D) møder forsøgspersonen til indgift af endotoxin. Forsøgsdagen forløber som forsøgsdag C, fraset at der ikke udføres DTPA-renografi eller muskel- og fedtvævsprøver.

Kosttilskuddet *Lactobacillus acidophilus* NCFM eller placebo indtages 2 gange dagligt i en periode på 4 uger, der indledes efter forsøget dag C og som afsluttes på forsøgsdag D. Forsøgspersonerne må ikke indtage surmælksprodukter under denne del af forsøget, men skal i øvrigt indtage deres vanlige kost.

Der skal i alt opsamles og indsendes fire friske afføringsprøver til Landbohøjskolen med henblik på at kunne påvise en evt. ændret sammensætning af tarmfloraen efter indtag af *Lactobacillus acidophilus* NCFM. Prøverne tages 7 dage forud for dag B, på dag B, på dag D og syv dage efter dag D.

*Bivikninger, ubehag og risici*.

Dropanlæggelsen giver gener svarende til en blodprøvetagning. Der tages i forbindelse med forsøget blodprøver til måling af signalstoffer, nyrefunktion og insulinfølsomhed; i alt ca. 300 ml over dag B og C samt 300 ml 4 uger efter sv.t. dag D og E. Dette indebærer ikke en risiko for forsøgspersonen.

Ved udtagelse af muskelvæv kan man ofte på trods af lokalbedøvelsen føle et sug eller en trykkende fornemmelse, sjældent en jagende smerte. Der kan sjældent opstå en blodansamling og yderst sjældent en mindre følefornemmelse, som forsvinder af sig selv igen. Fedtvævsprøven er udover anlæggelsen af lokalbedøvelsen ikke forbundet med betydende smerte.

Insulin-clamp med anvendelse af sukkersporstof er en sikker og velafprøvet metoder, der ikke er forbundet med gener ud over dropanlæggelserne.

Den totale stråledosis indgivet i forbindelse med DTPA-renografierne er godt 4 mSv, hvilket svarer til 2 års baggrundsstråling i Danmark. Man kan teoretisk beregne, at den indgivne strålemængde medfører en øget risiko for på længere sigt at dø af kræft på 0,02 %. Den samlede risiko for at dø af kræft øges herved fra de ca. 25 %, der gælder hele befolkningen, til 25,02 %.

Vi har tidligere anvendt endotoxin til raske forsøgspersoner med henblik på undersøgelse af immunresponset og stofskiftet. Endotoxin har endvidere talrige gange været anvendt til forsøg på raske frivillige i udlandet. Endotoxin er som nævnt et bakterieprodukt, men er ikke i sig selv infektiøst, dvs. at symptomerne forsvinder, når endotoxinet i løbet af nogle timer nedbrydes i kroppen. Vores hidtidige observationer af de kliniske fund hos forsøgspersonerne er i overensstemmelse med de i litteraturen beskrevne. Ved anvendelse af en lav dosis endotoxin (0,3 ng/kg) kan ses forbigående (timer) influenzalignende symptomer i form af hovedpine, kulderystelser, temperaturstigning, pulsstigning, muskelsmerter og/eller kvalme, mens blodtrykket er upåvirket. Der er registreret en kortvarig stigning i blodets indhold af forskellige signalmolekyler, men disse normaliseres igen inden for ca. 6-12 timer. Vi har, i overenstemmelse med litteraturen, ikke set alvorlige bivirkninger eller senfølger. Der er ikke tidligere givet endotoxin til type 2 diabetikere, men vi forventer ikke at den kliniske reaktion afskiller sig markant fra reaktionen hos raske forsøgspersoner. Alle forsøgspersoner observeres og monitoreres tæt, og der vil være en læge tilknyttet forsøgspersonerne uden andre opgaver end dette.

Der er ingen risici eller ubehag forbundet med indtag af *Lactobacillus acidophilus* NCFM.

### Støtte til projektet

Undersøgelsen af sammenhængen mellem nedsat insulinfølsomhed, inflammation og probiotiske bakteriers evt. helbredsfremmende effekt er finansieret af Det Strategiske Forskningsråd og indgår i et større samarbejde mellem Center for Inflammation og Metabolisme, Rigshospitalet, Landbohøjskolen (KVL), Danmarks teknologiske universitet og Danmarks pædagogiske universitet. KVL leverer *Lactobacillus acidophilus* NCFM samt placebo, som er fremstillet af Danisco. Til udførelse af DTPA-renografi m.h.p. bestemmelse af RPF/GFR i forbindelse med human endotoxinæmi er modtaget et legat på kr. 100.000,- fra Det Medicinske Selskab i København. Der søges om yderligere fondsstøtte fra offentlige og private fonde. Ud over, at Danisco er producent af probiotiske bakterier / placebo, er der ingen kommercielle interesser i eller støtte til projektet.

**Betændelsesreaktioner hos type 2 diabetikere – Betydning for nyrens blodtilførsel, insulinfølsomheden og effekt af mælkesyrebakterier.**

**-Information til forsøgspersoner.**

Vi henvender os til dig for at spørge, om du vil deltage i en videnskabelig undersøgelse. Deltagelse i undersøgelsen er frivillig, og et evt. tilsagn om deltagelse kan til enhver tid trækkes tilbage uden nogen form for konsekvenser. Undersøgelsen handler om akutte og kroniske irritationsreaktioner (såkaldt inflammation) hos type 2 diabetikere og raske, inflammations betydning for nyrernes blodtilførsel og for insulinfølsomheden. Endvidere vil vi undersøge effekten af et kosttilskud, mælkesyrebakterien *Lactobacillus acidophilus* NCFM, på intensiteten af inflammation og på følsomheden for insulin. (Forsøget er godkendt af De Videnskabetiske Komitéer for Københavns og Frederiksberg Kommuner, samt Den Videnskabsetiske Komité for Købenahavns Amt, journalnr. …)

På de næste sider vil vi beskrive, hvad forsøget går ud på, og hvordan du vil kunne deltage, såfremt du bestemmer dig for at ville være med. Det er frivilligt at deltage, og du vil på et hvilket som helst tidspunkt før og under undersøgelsen have ret til at ombestemme dig, uden at du skal komme med nogen forklaring på hvorfor. Det gælder også, selvom du har skrevet under på at ville deltage.

Vi har på de næste sider beskrevet forudsigelige bivirkninger og risici ved undersøgelsen. Ved en videnskabelig undersøgelse kan der også forekomme uforudsigelige bivirkninger og risici. Der vil derfor til stadighed være en læge til stede under forsøget for at overvåge og om nødvendigt behandle dig. Din deltagelse i forsøget vil blive afbrudt, hvis vi under forsøget registrerer resultater, der tyder på, at det ikke er sikkert for dig at fortsætte. Endvidere vil hele forsøget blive afbrudt, hvis vi observerer uforudsete risici, som vi ikke kan udsætte andre forsøgspersoner for. Det kan også blive nødvendigt at afbryde forsøget af tekniske årsager, fx hvis vi ikke kan anlægge de for undersøgelsen nødvendige katetre hos dig, eller hvis nødvendigt apparatur bryder sammen.

Du har ret til betænkningstid, før du evt. afgiver samtykke til at deltage. Udover denne skriftlige information vil du ligeledes modtage en mundtlig information om undersøgelsen, og her har du ret til at medbringe en bisidder. Det er også en god ide, at du læser den udleverede folder, ”Før du beslutter dig”, inden du bestemmer dig for, om du ønsker at deltage. Alle oplysninger om dine helbredsforhold er omfattet af tavshedspligt.

Du bør tage dig god tid til at læse alle papirerne, inden du beslutter dig. Hvis du vil deltage i undersøgelsen, bedes du underskrive samtykkeerklæringen og returnere den til os.

##### Forsøgets formål

Vi ønsker at undersøge om en eksperimentelt udløst inflammation / irritationsreaktion kan påvirke aktiviteten af bestemte gener i muskler og fedtvæv med betydning for insulinfølsomheden.

Vi vil undersøge nyrernes blodtilførsel og funktion under en eksperimentel irritationsreaktion. Nyrernes blodtilførsel og filtreringsegenskaber måles ved hjælp af DTPA-renografi (nyrefunktionsundersøgelse). Desuden undersøges nyrefunktionen ved en række blod- og urinprøver.

Vi vil undersøge om indtag af kosttilskuddet og mælkesyrebakterien *Lactobacillus acidophilus* NCFM i 4 uger kan ændre intensiteten af en eksperimentelt udløst irritationsreaktion.

Vi vil undersøge om insulinfølsomheden påvirkes ved daglig indtag af *Lactobacillus acidophilus* NCFM gennem en 4 ugers periode ved hjælp af en såkaldt insulin-clamp teknik og et sukkersporstof.

Endelig vil vi undersøge, om indtag af *Lactobacillus acidophilus* NCFM medfører en ændring i tarmfloraens sammensætning i afføringen.

Den eksperimentelt fremkaldte irritationsreaktion udløses ved at indgive en oprenset bakteriebestanddel (*E. coli* endotoxin) i blodbanen.

**Beskrivelse af forsøget:**

For at deltage skal du være mand og mellem 25 og 80 år gammel. Du skal enten være helt rask uden nogen form for kroniske sygdomme, eller også skal du lide af type 2 diabetes. Hvis du er diabetiker. må du ikke lide af hjerte-, lunge- eller nyresygdomme eller have andre pågående alvorlige lidelser, f.eks. symptomgivende åreforkalkning i benene. Hvis du er kendt med forhøjet blodtryk, skal dette være velreguleret. Du må maksimalt få 2 blodtrykssænkende midler, og disse må ikke være af ACE-hæmmer typen eller angiotensin II receptor antagonist typen, f.eks. corodil, captopril, odrik, cozaar m.v. Du må ejheller være i fast antibiotikabehandling. Du må ikke have været syg de sidste 14 dage før forsøget, og som rask forsøgsperson må du ikke tage nogen form for medicin ud over midler mod sæsonbetinget høfeber. Du må ikke være i behandling med blodfortyndende behandling med undtagelse af magnyl.

Hvis du tager magnyl, skal du pausere hermed 1 dag før forsøgsdag B og genoptage behandlingen dagen efter forsøgsdag C. Alle diabetespræparater og cholesterolsænkende lægemidler pauseres 1 uge forud for forsøgsdag B, og genoptages umiddelbart efter dag C. Præparaterne skal tilsvarende pauseres 1 uge før forsøgsdag D og genoptages efter dag E.

Pådrager du dig en febersygdom undervejs i forsøgsperioden, bedes du kontakte forsøgslederen med henblik på evt. ændring af datoerne for forsøgsdagene.

Forud for selve forsøget udføres en objektiv undersøgelse af dig. Der tages EKG (undersøgelse af hjertets elektriske aktivitet) og blodprøver. Disse undersøgelser skal sikre, at du ikke fejler andet end en eventuel allerede kendt type 2 diabetes. Efter forundersøgelsen vil det ved lodtrækning blive afgjort, om du skal have foretaget DTPA-renografi eller ej.

Forsøget løber over i alt 4-5 forsøgsdage, (A), B, C, D og E, fordelt over ca. 1½ måned. Forsøgsdag B og C samt forsøgsdag D og E udføres over 2 på hinanden følgende dage, adskilt af en 4 ugers periode.

På forsøgsdag A møder kun de forsøgspersoner skal have foretaget DTPA-renografi. Du møder på Klinisk fysiologisk afdeling med henblik DTPA-renografi til måling af en basismåling for nyrernes blodtilførsel og filtreringsegenskaber. Der vil blive anlagt et drop i hånden i forbindelse med undersøgelsen. Undersøgelsen varer inkl. forberedelser ca. 1 time.

På forsøgsdag B møder du fastende fra midnat på Center for Inflammation og Metabolisme, afsnit 7641, med henblik på udtagning af muskel- og fedtvævsprøver samt undersøgelse af insulinfølsomheden og sukkerstofskiftet. Forud for vævsprøverne anlægges lokalbedøvelse i huden. Herefter udtages med en større kanyle væv fra en stor muskel på lårets forside samt fedtvæv fra maven. Du får anlagt et drop i to armvener samt et drop i håndryggen. Efter udtagelsen af vævsprøver påbegyndes infusion med sukkersporstoffet. 2½ time senere opstartes insulin-clampen med løbende infusion af sukkervand og insulin, således dit blodsukker fastholdes svarende til fasteværdier hos raske. Der tages løbende blodprøver fra droppet i håndrygsvenen. Insulin-clampen afsluttes ved 13-tiden, og efter ca. 1 times yderligere observation af blodsukkeret, kan du tage hjem.

På forsøgsdag C skal du fastende fra midnat møde kl. 7 på Intensiv afdeling 4131, Rigshospitalet. Du får en seng at ligge i, bliver tilsluttet vores overvågningsudstyr og får lagt 2 drop i armvener. Du får kontinuerligt målt temperatur, EKG, puls, hjertets minutvolumen (mængden af blod, der pumpes ud pr. minut) og blodets iltmætning. Blodtryk registreres hver 15. minut.

Kl. 8 indgives endotoxin.

På de forsøgspersoner, der er udtrukket til DTPA-renografi, udføres renografi kl. 9.30 og kl. 14 på Klinisk fysiologisk afdeling.

Kl. 9, 11 og 15 udtages muskel- og fedtvævsprøver efter samme princip som forsøgsdag B. Blodprøver tages hver time umiddelbart inden og op til 8 timer efter indgift af endotoxin. Blodprøverne trækkes fra en af de anlagte drop. De første 8 timer efter indgift af endotoxin skal du hver time lade vandet i en kolbe med henblik på måling af den producerede mængde urin og urinundersøgelser. Der er stor erfaring med indgivelse af endotoxin til raske forsøgspersoner, og bivirkningerne er velkendte, se nedenfor.

Efter forsøgsdag C følger en 4 ugers periode, hvor der indtages 1 pakning *Lactobacillus acidophilus* NCFM eller et uvirksomt ”snydepræparat” uden Lactobaciller (placebo) 2 gange dagligt, dvs. morgen og aften. Det er ukendt for både dig og forsøgslederne, om du tilhører den gruppe, der indtager lactobaciller eller den gruppe, hvis pakninger indeholder placebo. De små pakninger med lactobaciller eller placebo udleveres ved periodens start fra Center for Inflammation og Metabolisme, og opbevares i køleskab indtil indtagelse. Ved indtagelse drysses pakningens indhold ud i et afkølet måltid. Du må i denne periode ikke indtage andre surmælkprodukter, dvs. ingen yoghurt, A-38, ymer, drikkeyoghurt m.m. eller paraghurt. Du skal i øvrigt tilstræbe at spise så tæt på din vanlige kost som muligt.

Den sidste dag i denne 4 ugers periode er forsøgsdag D, hvor du møder fastende fra midnat kl. 7 på Center for Inflammation og Metabolisme, afsnit 7641, med henblik på udførelse af insulin-clamp med sukkersporstof svarende til forsøgsdag B. Der tages ingen vævsprøver.

På forsøgsdag E møder du fastende fra midnat på Intensiv afdeling 4131 kl. 7 med henblik på endotoxinindgift. Forsøgsdagen forløber sv.t. dag C, dog udføres ikke DTPA-renografi, og der udtages ikke muskel- og fedtvævsprøver. Der tages ejheller urinprøver fra. Du forbliver indlagt i mindst 8 timer herefter.

Du skal opsamle i alt 4 afføringsprøver i løbet af forsøgsperioden og som skal indsendes til Landbohøjskolen med henblik på undersøgelse af tarmfloraens sammensætning. Prøverne skal tages 1 uge før dag B, på dag B, på dag D og 1 uge efter dag D. Nærmere information vedr. prøvetidspunkter samt mundtlig og skriftlig information vedr. selve prøveopsamlingen gives til forundersøgelsen.

##### Bivirkninger, ubehag og risici

Anlæggelse af de i forsøget anvendte drop svarer i gener til en blodprøvetagning. Efter fjernelse af droppene kan der forekomme en mindre lokal blødning og lokal ømhed af nogle dages varighed. I øvrigt er dropanlæggelsen risikofri.

I forbindelse med udtagelse af vævsprøver anlægges lokalbedøvelse, hvilket i gener svarer til anlæggelse af lokalbedøvelse hos tandlægen. Ved den efterfølgende udtagelse af muskelvæv kan man ofte på trods af lokalbedøvelsen føle et sug eller en trykkende fornemmelse, sjældent en jagende smerte. Der kan sjældent opstå en blodansamling og yderst sjældent en mindre føleforstyrrelse, som forsvinder af sig selv igen. Fedtvævsprøven er forbundet med minimale gener; der kan dog efterfølgende komme en blodansamling. Vævsprøverne udtages af erfaren læge.

Ud over anlæggelse af drop er insulin-clamp og indgift af sukkersporstoffet ikke forbundet med ubehag eller risici.

I forbindelse med udførelse af de i forsøget 3 planlagte DTPA-renografier anvendes radioaktive isotoper. Den samlede stråledosis herved er godt 4 mSv. Dette svarer til 2 år af den baggrundsstråling, som alle udsættes for i Danmark. Man kan teoretisk beregne, at den indgivne strålemængde medfører en øget risiko for på længere sigt at dø af kræft på 0,02 %. Den samlede risiko for at dø af kræft øges herved fra de ca. 25 %, der gælder hele befolkningen, til 25,02 %.

Bivirkninger ved indgift af endotoxin kan i den anvendte lave dosering være forbigående (timer) influenzalignende symptomer i form af hovedpine, kulderystelser, temperaturstigning, pulsstigning, muskelsmerter og/eller kvalme, mens blodtrykket er upåvirket. Der er aldrig beskrevet alvorlige bivirkninger, ej heller ved højere dosering. Endotoxin er ikke i sig selv infektiøst og nedbrydes hurtigt i kroppen. Endotoxin er ikke tidligere givet til type 2 diabetikere. Vi indgiver imidlertid en lav dosis endotoxin, og vi forventer ikke at at typen og graden af bivirkninger adskiller sig markant fra, hvad der ses hos raske forsøgspersoner. Indgiften af endotoxin finder sted under nøje overvågning og monitorering, og der vil konstant være en læge tilstede under hele forsøget.

Indtag af *Lactobacillus acidophilus* NCFM og opsamling af afføringsprøver er ikke forbundet med ubehag eller risici.

### **Støtte til projektet**

Undersøgelsen af sammenhængen mellem nedsat insulinfølsomhed, inflammation og probiotiske bakteriers evt. helbredsfremmende effekt er finansieret af Det Strategiske Forskningsråd og indgår i et større samarbejde mellem Center for Inflammation og Metabolisme, Rigshospitalet, Landbohøjskolen (KVL), Danmarks teknologiske universitet og Danmarks pædagogiske universitet. KVL leverer *Lactobacillus acidophilus* NCFM, som er fremstillet af Danisco, samt placebo. Til udførelse af DTPA-renografi m.h.p. bestemmelse af RPF/GFR i forbindelse med human endotoxinæmi er modtaget et legat på kr. 100.000,- fra Det Medicinske Selskab i København. Der søges om yderligere fondsstøtte fra offentlige og private fonde. Ud over, at Danisco er producent af *Lactobacillus acidophilus* NCFM, er der ingen kommercielle interesser i eller støtte til projektet.

Hvis du ønsker at deltage, bedes du underskrive vedhæftede samtykkeerklæring.

Med venlig hilsen

Sofie Andreasen Kirsten Møller Bente Klarlund Pedersen

Læge 1. reservelæge, ph.d. Professor, overlæge, dr. med.

Epidemiafdeling M Epidemiafdeling M Epidemiafdeling M

Center for Inflammation og Center for Inflammation og Center for Inflammation og

Metabolisme Metabolisme Metabolisme

Rigshospitalet Rigshospitalet Rigshospitalet

Tlf. 41 29 34 79 Tlf.35 45 75 45

/ 35 45 16 16

E-mail:

[sofie_andreasen@msn.com](mailto:sofie_andreasen@msn.com)

Jan Bonde, Overlæge, dr. med.

Intensiv terapi afsnit 4131, Rigshospitalet

Tlf. 35 45 82 05

**Endotoxinprojekt:**

**Betændelsesreaktioner hos type 2 diabetikere – betydning for nyrens gennemblødning, insulinfølsomheden og effekt af mælkesyrebakterier.**

**Samtykkeerklæring.**

Jeg er informeret om, at det er frivilligt at deltage, og om at jeg til enhver tid kan trække mit tilsagn om at deltage tilbage, uden at dette får nogen konsekvenser for mig.

Dato:

Underskrift:

Mundtlig og skriftlig information givet af læge

Dato:

Underskrift:

Reference List

1. Murray,C.J. & Lopez,A.D. Alternative projections of mortality and disability by cause 1990-2020: Global Burden of Disease Study. *Lancet* **349**, 1498-1504 (1997).

2. Leblanc,M. *et al.* Risk factors for acute renal failure: inherent and modifiable risks. *Curr. Opin. Crit Care* **11**, 533-536 (2005).

3. Petersen,A.M. & Pedersen,B.K. The anti-inflammatory effect of exercise. *J. Appl. Physiol* **98**, 1154-1162 (2005).

4. Wheeler,A.P. & Bernard,G.R. Treating patients with severe sepsis. *N. Engl. J. Med.* **340**, 207-214 (1999).

5. Angus,D.C. *et al.* Epidemiology of severe sepsis in the United States: analysis of incidence, outcome, and associated costs of care. *Crit Care Med.* **29**, 1303-1310 (2001).

6. Angus,D.C. *et al.* Epidemiology of severe sepsis in the United States: analysis of incidence, outcome, and associated costs of care. *Crit Care Med.* **29**, 1303-1310 (2001).

7. Parrillo,J.E. *et al.* Septic shock in humans. Advances in the understanding of pathogenesis, cardiovascular dysfunction, and therapy. *Ann. Intern. Med.* **113**, 227-242 (1990).

8. Angus,D.C. *et al.* Epidemiology of severe sepsis in the United States: analysis of incidence, outcome, and associated costs of care. *Crit Care Med.* **29**, 1303-1310 (2001).

9. De Backer,D., Creteur,J., Preiser,J.C., Dubois,M.J. & Vincent,J.L. Microvascular blood flow is altered in patients with sepsis. *Am. J. Respir. Crit Care Med.* **166**, 98-104 (2002).

10. Carton,J.A. *et al.* Diabetes mellitus and bacteraemia: a comparative study between diabetic and non-diabetic patients. *Eur. J. Med.* **1**, 281-287 (1992).

11. McCowen,K.C., Malhotra,A. & Bistrian,B.R. Stress-induced hyperglycemia. *Crit Care Clin.* **17**, 107-124 (2001).

12. Mizock,B.A. Metabolic derangements in sepsis and septic shock. *Crit Care Clin.* **16**, 319-36, vii (2000).

13. Moller,K. *et al.* Cerebral blood flow and oxidative metabolism during human endotoxemia. *J. Cereb. Blood Flow Metab* **22**, 1262-1270 (2002).

14. Moller,K. *et al.* Cerebral blood flow and oxidative metabolism during human endotoxemia. *J. Cereb. Blood Flow Metab* **22**, 1262-1270 (2002).

15. Krabbe,K.S. *et al.* Hypotension during endotoxemia in aged humans. *Eur. J. Anaesthesiol.* **18**, 572-575 (2001).

16. Krabbe,K.S. *et al.* Ageing is associated with a prolonged fever response in human endotoxemia. *Clin. Diagn. Lab Immunol.* **8**, 333-338 (2001).

17. Krabbe,K.S. *et al.* Activated T lymphocytes disappear from circulation during endotoxemia in humans. *Clin. Diagn. Lab Immunol.* **9**, 731-735 (2002).

18. Krabbe,K.S. *et al.* Low-dose endotoxemia and human neuropsychological functions. *Brain Behav. Immun.* **19**, 453-460 (2005).

19. Keller,P., Moller,K., Krabbe,K.S. & Pedersen,B.K. Circulating adiponectin levels during human endotoxaemia. *Clin. Exp. Immunol.* **134**, 107-110 (2003).

20. Johansen,J.S., Krabbe,K.S., Moller,K. & Pedersen,B.K. Circulating YKL-40 levels during human endotoxaemia. *Clin. Exp. Immunol.* **140**, 343-348 (2005).

21. Starkie,R., Ostrowski,S.R., Jauffred,S., Febbraio,M. & Pedersen,B.K. Exercise and IL-6 infusion inhibit endotoxin-induced TNF-alpha production in humans. *FASEB J.* **17**, 884-886 (2003).

22. Ostrowski,S.R. *et al.* Interleukin-6 infusion during human endotoxaemia inhibits in vitro release of the urokinase receptor from peripheral blood mononuclear cells. *Scand. J. Immunol.* **61**, 197-206 (2005).

23. Krogh-Madsen,R. *et al.* Effect of hyperglycemia and hyperinsulinemia on the response of IL-6, TNF-alpha, and FFAs to low-dose endotoxemia in humans. *Am. J. Physiol Endocrinol. Metab* **286**, E766-E772 (2004).

24. Bruunsgaard,H., Pedersen,A.N., Schroll,M., Skinhoj,P. & Pedersen,B.K. Impaired production of proinflammatory cytokines in response to lipopolysaccharide (LPS) stimulation in elderly humans. *Clin. Exp. Immunol.* **118**, 235-241 (1999).

25. Rangel-Frausto,M.S. *et al.* The natural history of the systemic inflammatory response syndrome (SIRS). A prospective study. *JAMA* **273**, 117-123 (1995).

26. Brivet,F.G., Kleinknecht,D.J., Loirat,P. & Landais,P.J. Acute renal failure in intensive care units--causes, outcome, and prognostic factors of hospital mortality; a prospective, multicenter study. French Study Group on Acute Renal Failure. *Crit Care Med.* **24**, 192-198 (1996).

27. Martin,G.S., Mannino,D.M., Eaton,S. & Moss,M. The epidemiology of sepsis in the United States from 1979 through 2000. *N. Engl. J. Med.* **348**, 1546-1554 (2003).

28. Brivet,F.G., Kleinknecht,D.J., Loirat,P. & Landais,P.J. Acute renal failure in intensive care units--causes, outcome, and prognostic factors of hospital mortality; a prospective, multicenter study. French Study Group on Acute Renal Failure. *Crit Care Med.* **24**, 192-198 (1996).

29. Leblanc,M. *et al.* Risk factors for acute renal failure: inherent and modifiable risks. *Curr. Opin. Crit Care* **11**, 533-536 (2005).

30. Schrier,R.W. & Wang,W. Acute renal failure and sepsis. *N. Engl. J. Med.* **351**, 159-169 (2004).

31. Camussi,G. *et al.* Lipopolysaccharide binding protein and CD14 modulate the synthesis of platelet-activating factor by human monocytes and mesangial and endothelial cells stimulated with lipopolysaccharide. *J. Immunol.* **155**, 316-324 (1995).

32. Kon,V. & Badr,K.F. Biological actions and pathophysiologic significance of endothelin in the kidney. *Kidney Int.* **40**, 1-12 (1991).

33. Klahr,S. Role of arachidonic acid metabolites in acute renal failure and sepsis. *Nephrol. Dial. Transplant.* **9 Suppl 4**, 52-56 (1994).

34. Lucas,C.E., Rector,F.E., Werner,M. & Rosenberg,I.K. Altered renal homeostasis with acute sepsis. Clinical significance. *Arch. Surg.* **106**, 444-449 (1973).

35. Rector,F., Goyal,S., Rosenberg,I.K. & Lucas,C.E. Sepsis: a mechanism for vasodilatation in the kidney. *Ann. Surg.* **178**, 222-226 (1973).

36. Brenner,M., Schaer,G.L., Mallory,D.L., Suffredini,A.F. & Parrillo,J.E. Detection of renal blood flow abnormalities in septic and critically ill patients using a newly designed indwelling thermodilution renal vein catheter. *Chest* **98**, 170-179 (1990).

37. Langenberg,C. *et al.* Renal blood flow in sepsis. *Crit Care* **9**, R363-R374 (2005).

38. Han,W.K. & Bonventre,J.V. Biologic markers for the early detection of acute kidney injury. *Curr. Opin. Crit Care* **10**, 476-482 (2004).

39. Roberfroid,M.B. Prebiotics and probiotics: are they functional foods? *Am. J. Clin. Nutr.* **71**, 1682S-1687S (2000).

40. O'Mahony,L. *et al.* Lactobacillus and bifidobacterium in irritable bowel syndrome: symptom responses and relationship to cytokine profiles. *Gastroenterology* **128**, 541-551 (2005).

41. Xiao,S.D. *et al.* Multicenter, randomized, controlled trial of heat-killed Lactobacillus acidophilus LB in patients with chronic diarrhea. *Adv. Ther.* **20**, 253-260 (2003).

42. Simakachorn,N. *et al.* Clinical evaluation of the addition of lyophilized, heat-killed Lactobacillus acidophilus LB to oral rehydration therapy in the treatment of acute diarrhea in children. *J. Pediatr. Gastroenterol. Nutr.* **30**, 68-72 (2000).

43. Parent,D. *et al.* Therapy of bacterial vaginosis using exogenously-applied Lactobacilli acidophili and a low dose of estriol: a placebo-controlled multicentric clinical trial. *Arzneimittelforschung.* **46**, 68-73 (1996).

44. Hallen,A., Jarstrand,C. & Pahlson,C. Treatment of bacterial vaginosis with lactobacilli. *Sex Transm. Dis.* **19**, 146-148 (1992).

45. Shalev,E., Battino,S., Weiner,E., Colodner,R. & Keness,Y. Ingestion of yogurt containing Lactobacillus acidophilus compared with pasteurized yogurt as prophylaxis for recurrent candidal vaginitis and bacterial vaginosis. *Arch. Fam. Med.* **5**, 593-596 (1996).

46. Faber,S., Rigden,S. & Lukaczer,D. The use of probiotics in the treatment of irritable bowel syndrome: two case reports. *Altern. Ther. Health Med.* **11**, 60-62 (2005).

47. Mustapha,A., Jiang,T. & Savaiano,D.A. Improvement of lactose digestion by humans following ingestion of unfermented acidophilus milk: influence of bile sensitivity, lactose transport, and acid tolerance of Lactobacillus acidophilus. *J. Dairy Sci.* **80**, 1537-1545 (1997).

48. Varcoe,J.J., Krejcarek,G., Busta,F. & Brady,L. Prophylactic feeding of Lactobacillus acidophilus NCFM to mice attenuates overt colonic hyperplasia. *J. Food Prot.* **66**, 457-465 (2003).

49. Varcoe,J. *et al.* Variable response to exogenous Lactobacillus acidophilus NCFM consumed in different delivery vehicles. *J. Appl. Microbiol.* **93**, 900-906 (2002).

50. Sui,J., Leighton,S., Busta,F. & Brady,L. 16S ribosomal DNA analysis of the faecal lactobacilli composition of human subjects consuming a probiotic strain Lactobacillus acidophilus NCFM. *J. Appl. Microbiol.* **93**, 907-912 (2002).

51. Altermann,E. *et al.* Complete genome sequence of the probiotic lactic acid bacterium Lactobacillus acidophilus NCFM. *Proc. Natl. Acad. Sci. U. S. A* **102**, 3906-3912 (2005).

52. Borriello,S.P. *et al.* Safety of probiotics that contain lactobacilli or bifidobacteria. *Clin. Infect. Dis.* **36**, 775-780 (2003).
